# Metabolic protein phosphoglycerate kinase 1 confers lung cancer migration by directly binding HIV Tat specific factor 1

**DOI:** 10.1038/s41420-021-00520-1

**Published:** 2021-06-05

**Authors:** Yu-Chan Chang, Ming-Hsien Chan, Chien-Hsiu Li, Chih-Jen Yang, Yu-Wen Tseng, Hsing-Fang Tsai, Jean Chiou, Michael Hsiao

**Affiliations:** 1 Deparment of Biomedical Imaging and Radiological Sciences, National Yang-Ming University, Taipei, Taiwan; 2 Deparment of Biomedical Imaging and Radiological Sciences, National Yang Ming Chiao Tung University, Taipei, Taiwan; 3 Genomics Research Center, Academia Sinica, Taipei, Taiwan; 4 Division of Pulmonary and Critical Care Medicine, Department of Internal Medicine, Kaohsiung Medical University Hospital, Kaohsiung Medical University, Kaohsiung, Taiwan; 5 Faculty of Medicine, College of Medicine, Kaohsiung Medical University, Kaohsiung, Taiwan; 6 Department of Biochemistry, College of Medicine, Kaohsiung Medical University, Kaohsiung, Taiwan

**Keywords:** Non-small-cell lung cancer, Tumour biomarkers, Cell migration

## Abstract

Phosphoglycerate kinase (PGK) is involved in glycolytic and various metabolic events. Dysfunction of PGK may induce metabolic reprogramming and the Warburg effect. In this study, we demonstrated that PGK1, but not PGK2, may play a key role in tumorigenesis and is associated with metastasis. We observed an inverse correlation between PGK1 and the survival rate in several clinical cohorts through bioinformatics statistical and immunohistochemical staining analyses. Surprisingly, we found that PGK1 was significantly increased in adenocarcinoma compared with other subtypes. Thus, we established a PGK1-based proteomics dataset by a pull-down assay. We further investigated HIV-1 Tat Specific Factor 1 (HTATSF1), a potential binding partner, through protein–protein interactions. Then, we confirmed that PGK1 indeed bound to HTATSF1 by two-way immunoprecipitation experiments. In addition, we generated several mutant clones of PGK1 through site-directed mutagenesis, including mutagenesis of the N-terminal region, the enzyme catalytic domain, and the C-terminal region. We observed that even though the phosphoglycerate kinase activity had been inhibited, the migration ability induced by PGK1 was maintained. Moreover, our immunofluorescence staining also indicated the translocation of PGK1 from the cytoplasm to the nucleus and its colocalization with HTATSF1. From the results presented in this study, we propose a novel model in which the PGK1 binds to HTATSF1 and exerts functional control of cancer metastasis. In addition, we also showed a nonenzymatic function of PGK1.

## Introduction

Metabolic reprogramming is considered a hallmark in cancer research^1^. However, it is necessary to understand the mechanisms of each gene of metabolism during cancer progression. Glycolysis plays an initial role in the metabolic network, which controls the carbohydrate products and synthesis of downstream side-chain products (including the pentose phosphate pathway, amino acid and fatty acid biosynthesis)^2^. Glycolytic enzymes are an aspect that needs more attention in cancer research. Previous reports indicated that most of the glycolytic enzymes showed an aberrant status in tumorigenesis^3^. The most important thing is that the aberrant expression of glycolytic enzymes also affects the intracellular conditions and the remodeling tumor microenvironment, including hypoxia, acidic state, and chemokines accumulation^4–6^. Therefore, several studies have been conducted to identify promising glycolytic enzymes or metabolic pathways through proteomic or high-throughput biochemical strategies to identify novel biomarkers for diagnosis or for pharmacological targets for cancer therapy^3,7^. Although several inhibitors of glycolytic enzymes have been developed as anti-cancer agents, their effect are not obvious, strong side-effects and metabolic homeostasis is disturbed^8^. These glycolytic enzymes still need to investigate their role in tumorigenesis.

Phosphoglycerate kinase (PGK), a family of glycolytic enzymes that catalyze the reversible conversion of 1,2-bisphosphoglycerate to 3-phosphoglycerate, is composed of PGK1 and PGK2^9^. PGK1 is a ubiquitous enzyme in all somatic cells and PGK2 is preferentially expressed in the testis^10^. Notably, PGK1, which is highly conserved from rabbits to humans and is a well-studied isozyme of the PGK family, has been reported to be upregulated in various types of tumor cells, such as colorectal cancer^11,12^, breast^4^, hepatocellular carcinoma^13^, gallbladder cancer^14^, and lung cancer. In addition, dysfunction of the PGK family involves metabolic programming and the Warburg effect in tumorigenesis^15^. The uptake of intermediates producing lactic acid at the tumor site increases in the tumor. Nevertheless, the essential role of PGK1 in cancer development remains unclear. How PGK1 interacts with these pathways to control glucose metabolism during cancer progression is worth exploring.

Here, we demonstrated that high level of PGK1 protein was correlated with patient survival, both overall survival and disease-free survival, whereas PGK2 was not. Moreover, we identified that the PGK1 protein translocated from the cytoplasm to the nucleus in malignant cancer cells and advanced patients. Therefore, we established a PGK1-based proteomics dataset and found that the FUS RNA binding protein and the HIV-1 Tat Specific Factor 1 (HTATSF1) were binding partners through a protein–protein interaction (PPI). Taken together, the results of this study indicate that, we have identified a key metabolic gene, PGK1, which is critical for supporting cancer diagnosis and therapy. These results also provide a novel strategy against cancer metastasis in lung cancer.

## Methods

### Case selection

A total of 111 patients, diagnosed with non-small-cell lung cancer at the Kaohsiung Medical University Hospital of Taiwan from 1991 to 2007, were included in this study (KMUH-IRB-2011-0286). Patients who received preoperative chemotherapy or radiation therapy were excluded. Clinical information and pathology data were collected via a retrospective review of the medical records. All cases were staged according to the 7th edition of the Cancer Staging Manual of the American Joint Committee on Cancer (AJCC) and the histological cancer type was classified according to the World Health Organization (WHO) 2004 classification. Follow-up data were available in all cases, and the longest clinical follow-up time was 190 months. Overall survival (OS) and disease-free survival (DFS) were defined as the interval from surgery to death caused by the non-small-cell lung cancer and the recurrence of distant metastasis, respectively. The study was carried out with the approval of the Institutional Review Board and the permission of the ethics committee of the institution involved.

### Immunohistochemistry analysis

Immunohistochemical (IHC) staining was performed on serial formalin-fixed, paraffin-embedded tissue sections (5 μM) from the tissue microarray (TMA) using an automated immunostainer (Ventana Discovery XT Autostainer, Ventana Medical Systems, Tucson, AZ). The sections were first dewaxed in a 60 °C oven, after which they were deparaffinized in xylene, and then rehydrated. The antigens were retrieved by heat induced antigen retrieval for 30 min in TRIS-EDTA buffer. The slides were stained with the polyclonal rabbit antihuman antibodies against PGK1 (1:500, GeneTex, Hsinchu, Taiwan) and HTATSF1 (1:100, Proteintech, Rosemont, IL, USA). The sections were subsequently counterstained with hematoxylin, dehydrated, and mounted.

### TMA immunohistochemistry interpretation

The IHC staining assessment was independently conducted by two pathologists who were blinded to patient outcome. Only cytoplasmic expression of tumor cells in the cores were evaluated. Both the immunoreactivity intensity and the percentage were recorded. The intensity of staining was scored using a four-tier scale and defined as follows: 0, no staining; 1+, weak staining; 2+, moderate staining; and 3+, strong staining. The extent of staining was scored by the percentage of positive cells (0–100%). The final IHC scores (0–300) were obtained by multiplying the staining intensity score by the percentage of positive cells. All cases were divided into two groups according to the final IHC scores. High IHC expression level was defined as a score ≥150, and a score <150 was defined as low expression.

### Cell culture and materials

Human lung adenocarcinoma cell lines, (H1355, H1573, H23, A549, H1975, and H441) were purchased from the American Type Culture Collection (ATCC) cell bank. The lung adenocarcinoma cell lines CL1-0 and CL1-5 were established and provided as a gift from Dr. Pan-Chyr Yang (National Taiwan University, Taipei, Taiwan). H1355, H1573, H23, CL1-0, H1975, H441, and CL1-5 cells were maintained in RPMI 1640 medium, supplemented with 10% fetal bovine serum (FBS) (Invitrogen, Carlsbad, CA, USA). The human lung adenocarcinoma cell line A549 was grown in F12K medium plus 10% FBS (Invitrogen) at 37 °C in a humidified atmosphere of 5% CO_2_. For establishing the stable PGK1 or HTATSF1 cell lines, the pGIPZ lentiviral shRNAmir system (Thermo, Waltham, MA, USA) was used with PGK1 and HTATSF1 sequences, respectively. The lentiviruses were used to infect CL1-5 cells for 2 days. Stable clones were selected with puromycin (Sigma-Aldrich, St. Louis, MO, USA) at 1 μg/ml for 2 weeks.

### RNA extraction and site-directed mutagenesis analysis

Total RNA was extracted using the Trizol reagent according to the manufacturer’s protocol. The amount of RNA was measured using a NanoDrop spectrophotometer (Thermo, Waltham, MA, USA) and 5 μg of total RNA was used for cDNA synthesis for further experiments. According to the manufacturer’s protocol, the GeneArt kit (Invitrogen, Carlsbad, CA, USA) is used for site-directed mutagenesis analysis. The sequences of the primers are listed in the key resource table.

### Gene construction and lentivirus production

Lentiviral envelope and packing plasmid (pMDG and pΔ8.91) were purchased from the National RNAi core facility (Academia Sinica, Taiwan). PGK1 lentiviral shRNA constructs and pGIPZ nonsilence, a nonsilence shRNA construct were purchased from CLONTECH (USA). Lentiviruses were cotransfected into 293T cell with pMDG, pΔ8.91, and the shRNA construct using a calcium phosphate transfection method. After 48 h of incubation, lentiviruses were collected and used to infect the cells with polybrene (2 μg/ml). The cells with altered PGK1 expression were selected with puromycin (2 μg/ml) for 1 week. Full length PGK1 cDNAs were amplified from the MGC gene bank (Open Biosystem Inc., from Dr. Michael Hsiao’s library) by using PCR. The cDNAs were first cloned into a pENTR1A vector (Gateway pENTR 1A Dual Selection Vector), then subcloned into pLenti6.3/V5-DEST. The PGK1 overexpression cells with altered PGK1 expression were selected with blasticidin (2 μg/ml) for 1 week.

### PGK1 enzyme activity

Phosphoglycerate kinase activity was measured using colorimetric phosphoglycerate kinase assay kits (BioVision, Milpitas, CA, USA) according to the manufacturer’s protocol. Briefly, cells from the designed experiments were incubated with assay buffer containing ATP, NADH, substrate, and developer. Then, the optical densities were measured at 340 nm wavelengths.

### Cell migration assay

The cells were seeded on fibronectin-coated polycarbonate membranes in Boyden’s chambers and maintained in serum-free medium. After a suitable time, the cells on lower side of the membrane were stained and counted under a light microscope (×400, 8 random fields from each well). The average number of cells and the SD were calculated based on quadruplicate experiments.

### Western blotting

The cells were lysed in RIPA buffer for 30 min followed by centrifugation at 13,000 rpm for 15 min at 4 °C. The protein concentration was measured using BCA protein assay reagents (Thermo, Waltham, MA, USA). Total proteins (30 μg) were separated by SDS-PAGE on 10% polyacrylamide gels and transferred onto PVDF membranes. The membranes were incubated with primary antibodies overnight after blocking in 5% nonfat milk for 30 min. Secondary antibodies were incubated for 1 h, after which the proteins were visualized using enhanced chemiluminescence (ECL) reagents (Perkin Elmer, Waltham, MA, USA). Quantitative data were obtained using ImageJ software.

### Immunoprecipitation and immunoblotting analysis

Whole cell lysates (2 mg) from the cultured cells were incubated overnight in IP buffer with 25 μl of protein A/G magnetic beads and corresponding antibodies against PGK1 (1:100) (GTX7614, GeneTex, Hsinchu, Taiwan) and HTATSF1 (4 μg) (Cat# No. 20805-1-AP, Proteintech, Rosemont, IL, USA) in a 1.5-ml microcentrifuge tube with a final volume of 1000 μl. Proteins-interacting with the antibodies were purified according to the manufacturer’s protocol.

### Statistical analysis

Estimates of the survival rates were analyzed by the Kaplan–Meier method and compared by the log-rank test. The follow-up time was censored if the patient was lost during follow-up. Statistics analysis was performed with SPSS 17.0 software (SPSS, Chicago, IL, USA). A paired t-test was performed to compare the PGK1/HTATSF1 IHC expression in cancer tissue and in the corresponding normal mucosal tissue. Univariate and multivariate analyses were performed using a Cox proportional hazards regression analysis with and without an adjustment for PGK1 in combination with HTATSF1, IHC expression level, tumor stage, lymph node stage, and metastasis. For all analyses, a *p* value of <0.05 was considered statistically significant.

## Results

### The up-regulation of the PGK1 mRNA level predicts poor clinical outcomes in lung cancer patients

**Fig. 1:**
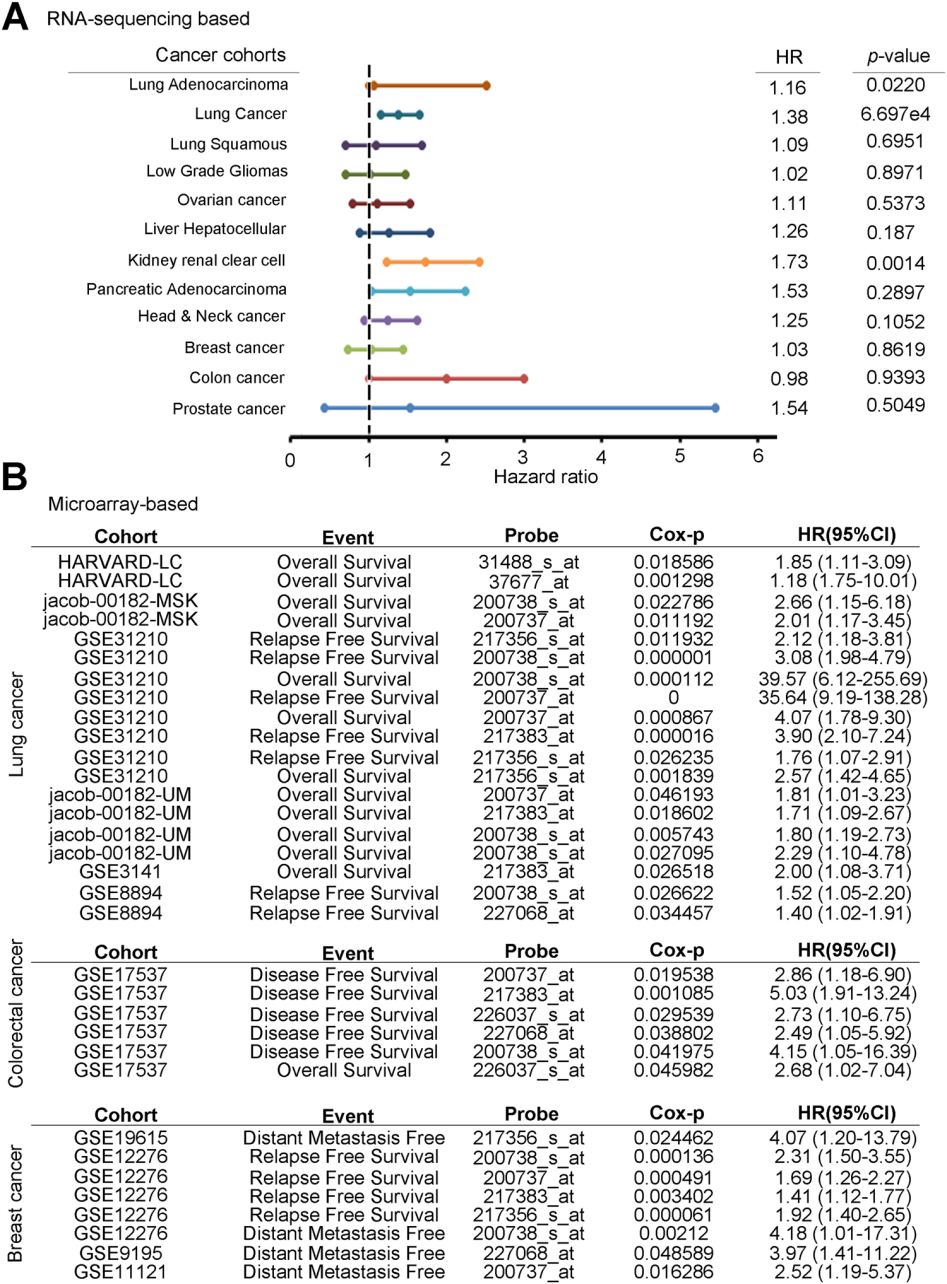
PGK1 serves as an independent prognostic factor for various cancer patients. **A** Survival analysis of RNA sequencing data from TCGA cohort showed that correlation with hazard ratio and *P*-value of PGK1 in several malignant cancer types included lung, glioblastoma, ovarian, liver kidney, pancreas, head and neck, breast, colon and prostate cancer. **B** A global meta-analysis of PGK1 gene expression using the PrognoScan database. The hazard ratio (HR) at a 95% confidence interval (CI) is shown and is accompanied by the range of HR values from lowest to highest in a forest plot. The significance of the difference was analyzed using the nonparametric Mann–Whitney *U* test.

**Fig. 2:**
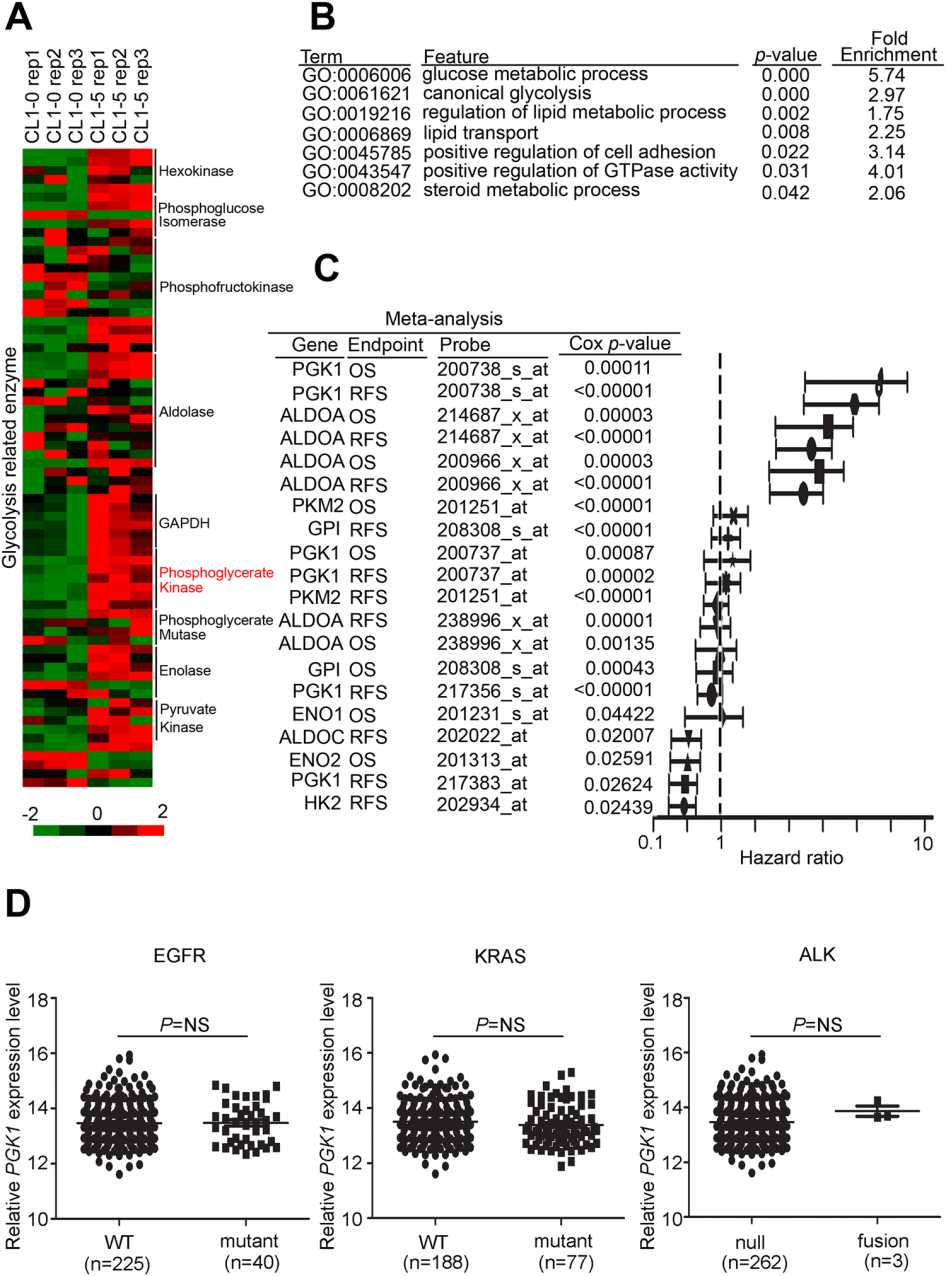
PGK1 expression is correlated with cancer metastasis in lung cancer patients. **A** Heat-map of the microarray results (GSE42407) of the mRNA expression of glycolytic enzyme in CL1-5 lung cancer cells compared to CL1-0 cells. **B** KEGG predicts potential canonical pathways and gene annotation by >2.0-fold-change in CL1-5 cells. **C** A global meta-analysis of expression of several glycolytic genes using the PrognoScan database. The hazard ratio (HR) at a 95% confidence interval (CI) is shown and is accompanied by the range of HR values from lowest to highest in a forest plot. **D** Quantification of PGK1 expression by in-silico analysis of lung cancer patients by each corresponding clinical event (EGFR mutant, KRAS mutant, and ALK fusion status).

In each cancer, the production of metabolites and metabolic reprogramming have different degrees. The expression of glycolytic enzymes also changed accordingly. To understand which cancers of PGK1 will seriously affect the prognosis of patients, so we have comprehensively screened the clinicopathological factors of PGK1 on patient survival rate. We inputted the TCGA dataset and multiple microarray-based clinical cohorts, and both found that PGK1 had the most significant potential in lung cancer (Fig. 1). In order to confirm that only PGK1 is aberrantly expressed or a variety of glycolytic enzymes are aberrantly expressed. We examined the relevance of glycolysis-related gene expression to cancer metabolism in lung cancer, we performed a large-scale transcriptomics analysis of microarray data from the Gene Expression Omnibus (GEO) microarray dataset (GSE42407) that was focused on glycolytic enzymes. The datasets include a triple repetition of the CL1-5 lung cancer cell line and its counterpart cell line, CL1-0. CL1-0, and CL1-5, have been isolated and artificially generated through a transwell invasion chamber method by the CL1-1, CL1-2, CL1-3, CL1-4, and CL1-5 of series adenocarcinoma sublines^16^. CL1-5 has a mesenchymal-type morphology, a high metastatic ability and multiple oncogenic pathways are activated. Mesenchymal-type markers *SNAI1*, *SNAI2* and *TWIST1* were detected in CL1-5, and *CDH1* was inhibited in CL1-5 cells. In addition, the CL1-0 and CL1-5 cells have been used to establish several omics-based datasets, including transcriptomics^17^, microRNAs^18^, secretomics^19^, and proteomics. Furthermore, characterization and metabolic reprogramming of glycolysis-related enzymes have been mentioned as being implicated in tumorigenesis^20^. After normalization, our results revealed that most of the glycolytic enzymes had been upregulated in CL1-5 compared with CL1-0 with a 1.5-fold-change cut-off signature, especially the phosphoglycerate kinase family (Fig. 2A and Supplementary Table 1). We also utilized the signature to predict potential canonical pathways with the gene ontology tool. The results showed that several glycolysis, lipid synthesis, and metastasis panels were found in our transcriptomics profile (Fig. 2B). We further performed a global prognostic meta-analysis for glycolysis-related genes against the microarray data from clinical cohorts with lung cancer using the PrognoScan database. The meta-analysis showed that the up-regulation of PGK1 significantly correlated with a poorer prognosis as shown by the increased hazard ratio in patients (Fig. 2C). Moreover, we determined the expression of PGK1 with several typical genetic alteration events in lung cancer, including EGFR mutants, KRAS mutants, and ALK fusions in The Cancer Genome Atlas (TCGA) clinical cohort. However, our data showed that PGK1 expression did not correlate with genetic alterations in lung cancer (Fig. 2D).

**Fig. 3:**
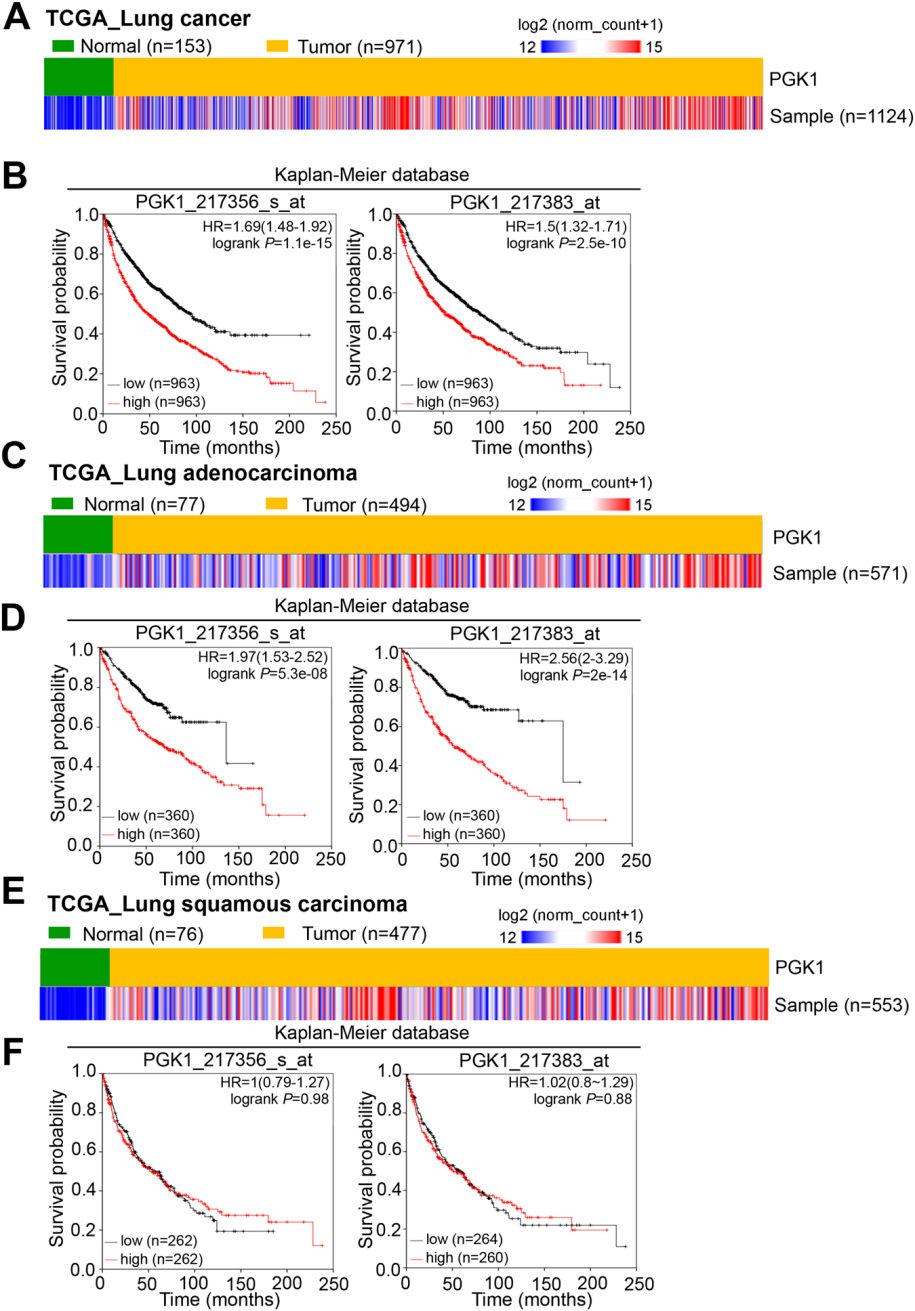
PGK1 expression is correlated with survival time in lung cancer. **A** Heat-map of PGK1 gene expression from the TCGA RNA sequence database in nontumor and tumor tissues derived from clinical patients with lung cancer. **B** Kaplan–Meier analysis of PGK1 RNA expression at concurrently low or high levels by in-silico analysis at the endpoint of overall survival probability in TCGA lung cancer patients (*p* = 1.1e-15, *p* = 2.5e-10, respectively). **C** Heat-map of PGK1 gene expression from the TCGA RNA sequence database in nontumor and tumor tissues derived from clinical patients with lung adenocarcinoma. **D** Kaplan–Meier analysis of PGK1 RNA expression at concurrently low or high levels by in-silico analysis at the endpoint of overall survival probability in TCGA lung adenocarcinoma patients (*p* = 5.3e-08, *p* = 2e-14, respectively). **E** Heat-map of PGK1 gene expression from the TCGA RNA sequence database in nontumor and tumor tissues derived from clinical patients with lung squamous carcinoma. **F** Kaplan–Meier analysis of PGK1 RNA expression at concurrently low or high levels by in-silico analysis at the endpoint of overall survival probability in TCGA lung squamous carcinoma patients (*p* = 0.98, *p* = 0.88, respectively).

Among the different datasets, the lung cancer patients with PGK1 overexpression had a significantly reduced survival probability, suggesting that higher PGK1 expression likely contributed to the mechanisms of cancer progression, such as chemotherapy resistance and metastasis. We further examined the gene expression of *PGK1* in lung cancer patients by analyzing the online TCGA database and the GEO meta-analysis cohorts. According to the validation, we observed a higher *PGK1* expression in lung cancer samples compared to adjacent normal tissues (Fig. 3A, B). Consistently, the data from the microarray analysis (GSE7670) from GEO definitively demonstrated that *PGK1* gene expression in the tumor portions was extensively higher than that in the paired normal adjacent tissues in lung cancer patients (Supplementary Fig. 1). We further validated *PGK1* expression with two different probes (217356_s_at and 217383_at) in the lung cancer cohort from the Kaplan–Meier Plotter website. The data suggested that the overexpression of *PGK1* was strongly associated with a poor overall survival and a progression-free survival in lung cancer, especially in the adenocarcinoma subtype, which has been thought to be the most common type of lung cancer clinically (Fig. 3C–F). Although PGK2 is in the same family, it also showed consistent trends in the survival curve in lung cancer and lung adenocarcinoma patients. However, PGK2 was rarely expressed in clinical specimens (Supplementary Figs. 2–3). Therefore, we further investigated the role of PGK1 in lung adenocarcinoma.

### Expression of PGK1 is correlated with migration ability in lung adenocarcinoma

**Fig. 4:**
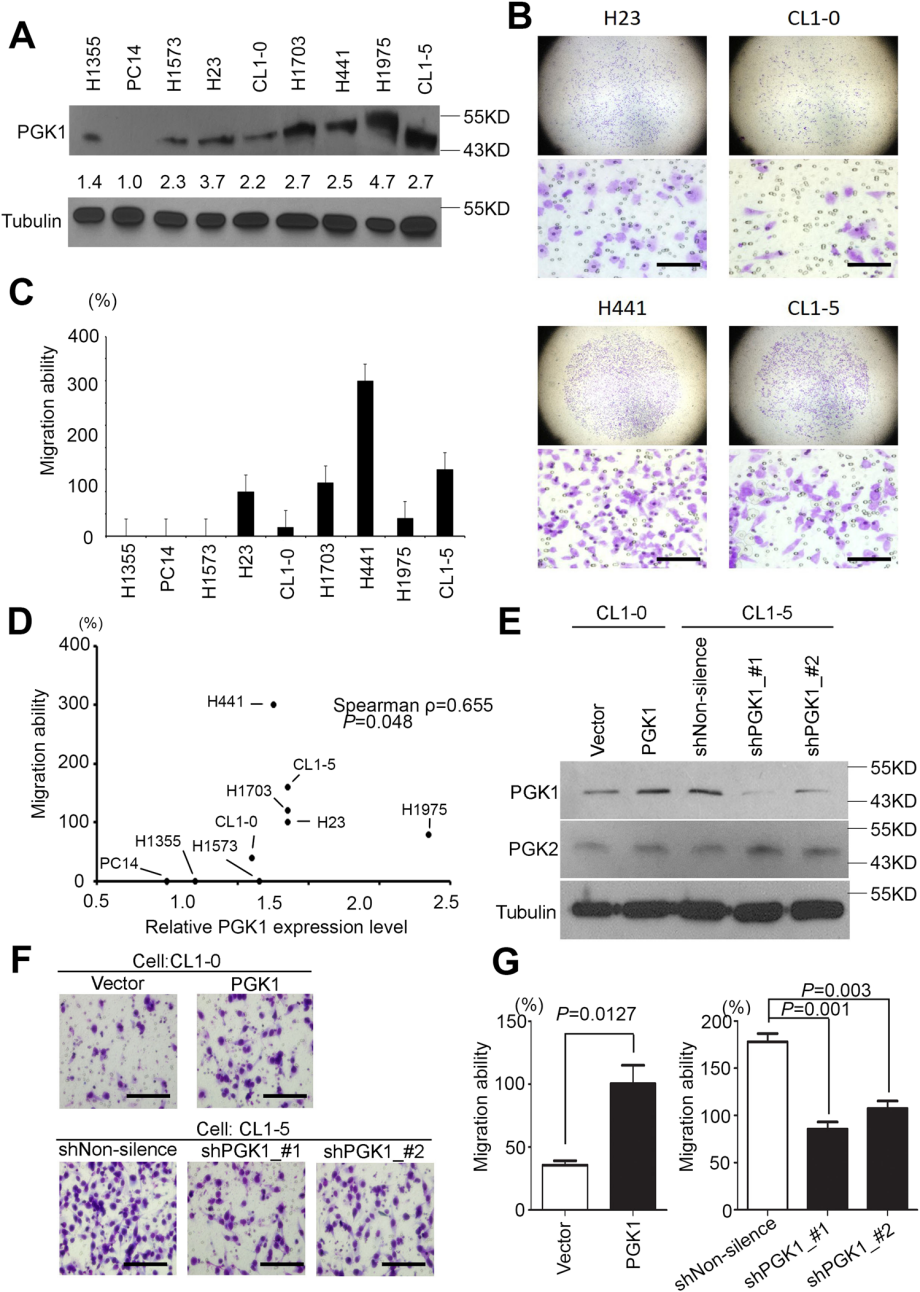
PGK1 regulates cancer migration in lung cancer. **A** Western blot analyses for endogenous expression of PGK1 in various lung adenocarcinoma cells. Tubulin was used as the internal control for protein loading. **B** Representative Giemsa staining of several lung adenocarcinoma cells including H23, CL1-0, H441, and CL1-5, after migration through a Boyden’s chamber. Scale bar: 100 μm. **C** Quantification of the migration ability of the lung adenocarcinoma cells panel. **D** The correlation of PGK1 expression and the metastatic lung cancer cell lines was analyzed by the nonparametric Spearman method. **E** The determination of PGK1 and PGK2 protein levels in CL1-0 cells with vector control or with PGK1 overexpression. The determination of PGK1 protein levels in CL1-5 cells after transfection with nonsilencing and PGK1 shRNAs. **F** Migration ability of the PGK1-overexpressing stable clone of CL1-0 cells was determined compared to the vector control stable clone (upper). Migration ability of the PGK1 knockdown stable clones of CL1-5 cells was determined, compared to the nonsilencing shRNA control stable clone (lower). Scale bar: 100 μm. **G** Quantitation the migration ability of the PGK1 knockdown models. The migration activities of the CL1-5 cells transfected with the designated shRNA clones of PGK1 (bottom). The data from three independent experiments are shown as the mean ± SD. The symbol ***p < 0.001 in the nonparametric Mann–Whitney test.

We collected and established a lung adenocarcinoma cell panel for screening the endogenous PGK1 protein levels. Quantification of the protein level was conducted using western blots. We observed that CL1-5, H23, and H441 had high expression levels compared to the others cell lines (Fig. 4A). We also calculated the migration ability through a Boyden’s chamber for lung adenocarcinoma cells (Fig. 4B). We ensured that the PC14 cells could not access the membrane. Therefore, our data showed that H23, H1073, H441, and CL1-5 cells had a high migration ability compared to the other cell lines (Fig. 4C). We further statistically analyzed the PGK1 protein expression in relation to migration ability, and the results showed that they were positively correlated in various lung adenocarcinoma cells (Fig. 4D, Pearson *ρ* = 0.683, *p* = 0.043). Similarly, the RNA level from in-silico analyses with next-generation sequencing assays showed PGK1, rather than PGK2, overexpression in several malignant lung cancer cells (Supplementary Fig. 4). Thus, we chose CL1-5 to establish a PGK1 knockdown model with two independent shRNA clones and CL1-0 for our PGK1 overexpression model. We validated the PGK1 protein level in each cell model by western blots (Fig. 4E). Furthermore, we determined the migration ability in available PGK1 overexpression and knockdown models (Fig. 4F and Supplementary Fig. 5). Taken together, these results indicated that we identified the characteristics of PGK1 as the key enzyme for promoting lung cancer migration, especially in the adenocarcinoma subtype (Fig. 4G).

### HTATSF1 is one of the binding partners of PGK1 in the lung adenocarcinoma migration model

**Fig. 5:**
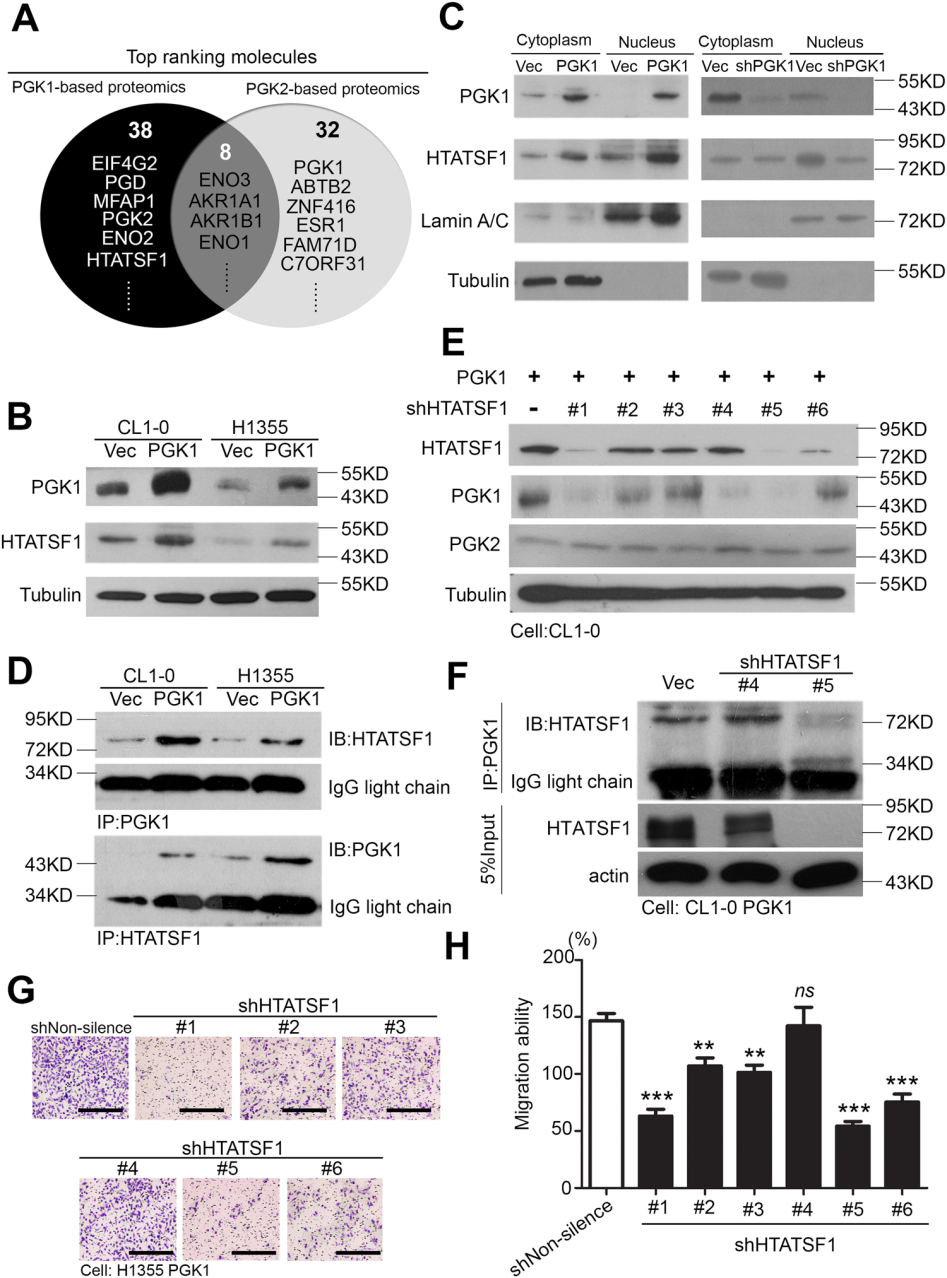
PGK1 directly binds to HTATSF1 via protein–protein interactions and promotes migration. **A** The molecules that indicated to interact in the PGK1- or PGK2-based proteomics profiles according to BioGrid website analysis. **B** Western blot analysis of PGK1 and HTATSF1 expression from CL1-0 and H1355 cells with the forced expression of the vector control or exogenous PGK1 gene. **C** Western blot analysis of PGK1 and HTATSF1 protein levels in the nuclear protein fraction and cytoplasmic fraction derived from PGK1 two-way models. **D** Pull-down assay for whole-cell lysates derived from CL1-0 and H1355 cells with the forced expression of PGK1 or vector control using beads followed by western blot analysis of the HTATSF1 and PGK1 proteins. **E** Western blot analysis of HTATSF1, PGK1 and PGK2 expression from CL1-0 cells with or without the shHTATSF1 clones. **F** Pull-down assay for whole cell lysates derived from CL1-0 PGK1 cells with or without the shHTATSF1 clones using beads followed by western blot analysis of HTATSF1 proteins. **G** Representative Giemsa staining to estimate the migration abilities of the H1355 PGK1 cells transfected with the designated shRNA clones of the HTATSF1 gene. Scale bar: 100 μm. **H** Cellular migration abilities of the H1355 PGK1 cells transfected with the designated shRNA clones of the HTATSF1 gene. The symbols **p < 0.01 and ***p < 0.001 in the nonparametric Mann–Whitney test.

The expression level of PGK1 has been studied in relation to patients’ survival curves and several phenotypes in tumorigenesis. However, the detailed mechanisms and interatomic processes are still unknown. We attempted to identify several interaction partners of PGK1 from the latest references on the BioGrid website. Previous studies have shown that PGK1 and PGK2 have 88% similar amino acid sequence. For this situation, we excluded many of the molecules available in both PGK1 and PGK2 interatomic datasets (Supplementary Table 2) to decide which molecules had specific affinity for PGK1. In previous studies, we confirmed that PGK1 can control the migration ability of lung cancer cells (Fig. 4). Therefore, we evaluated the hazard ratio in the clinical population of each candidates and measured the migration capacity in the shRNA clones (Supplementary Figs. S6–S7). HTATSF1 was revealed as a top ranking factor in the PGK1 interactionomics (Fig. 5A). We then verified the expression of several targets in the PGK1 overexpression models. The results showed that the HTATSF1 protein levels were elevated in the cell models (Fig. 5B). In addition, we observed that PGK1 translocated in the nuclear position and coordinated with HTATSF1 (Fig. 5C). Furthermore, we observed the protein-protein interactions (PPI) in these models. Through a two-way model, we confirmed that PGK1 and HTATSF1 could directly bind in lung cancer cells (Fig. 5D and Supplementary Fig. S8). We then performed a knockdown of HTATSF1 by shRNAs in the PGK1 overexpression model, and the results showed that the migration ability was inhibited depending on the knockdown efficiency of HTATSF1 (Fig. 5E–H). This evidence supports the hypothesis that the PPI between PGK1 and HTATSF1 may promote lung cancer metastatic ability. Taken together, these results lead us propose that PGK1 coordinates with HTATSF1 to promote lung cancer migration through protein–protein interactions.

**Fig. 6:**
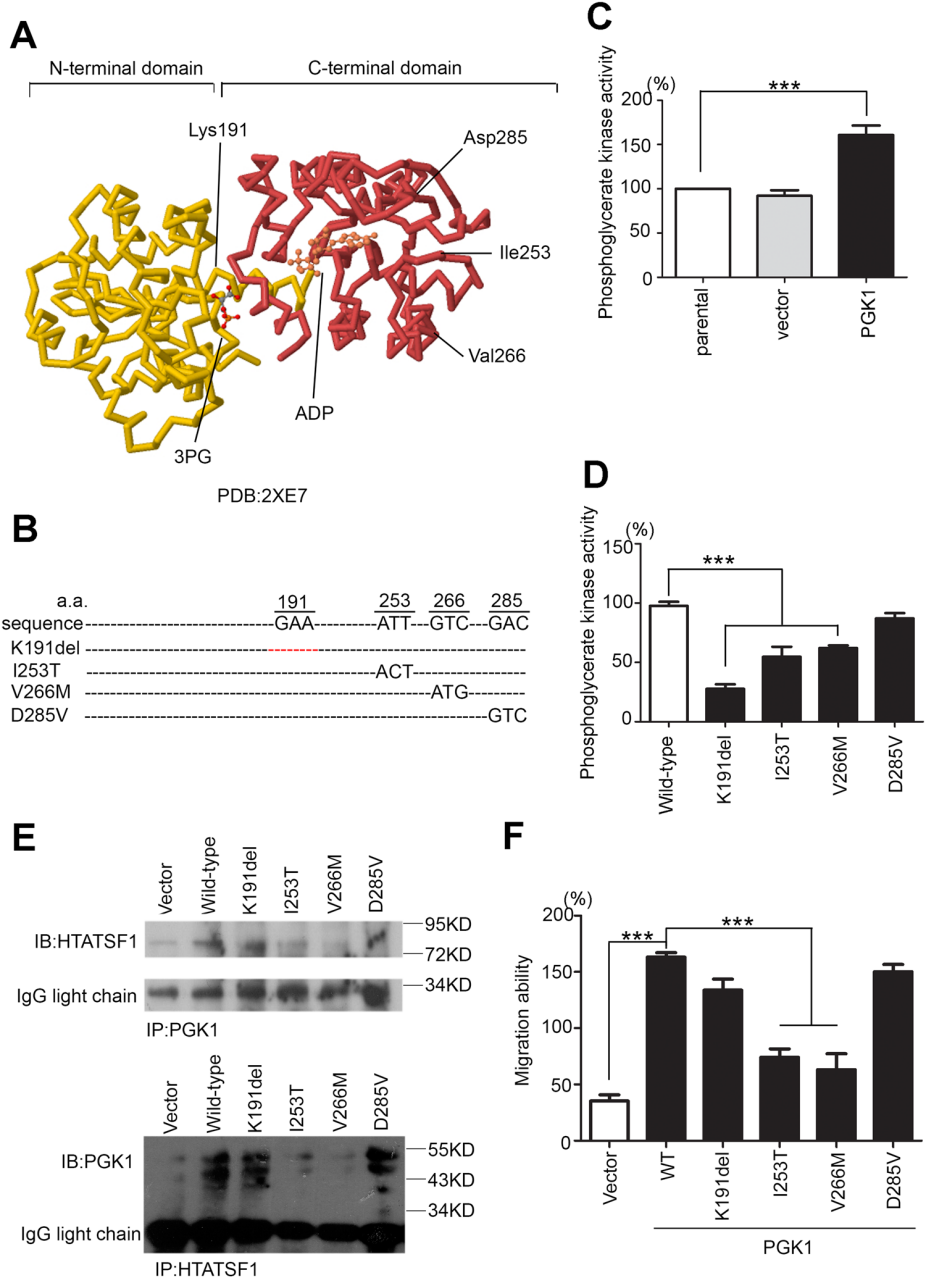
The binding affinity of PGK1-HTATSF1 reflects the lung cancer migration ability. **A** The crystal structure of PGK1 includes two domains and a 3-PG/ADP binding site (PDB ID:2XE7). **B** Sequences of the amino acids of the PGK1 wild-type gene, primer and several mutant forms that were designed. **C** Intracellular phosphoglycerate kinase activity of the PGK1 overexpression model. **D** Intracellular phosphoglycerate kinase activity in H1355 cells after the forced expression of exogenous wild-type or mutant PGK1 genes. **E** Pull-down assay for whole cell lysates derived from H1355 cells with the forced expression of exogenous wild-type (wt) or mutant PGK1 using beads followed by western blot analysis of the HTATSF1 and PGK1 proteins. **F** Cellular migration abilities of H1355 cells after the forced expression of exogenous wild-type or mutant PGK1. The symbol ***p < 0.001 in the nonparametric Mann–Whitney test.

Previous studies have shown that most glycolytic enzymes may undergo metabolic reprogramming of their enzymatic function during tumorigenesis^21^. For these purposes, we examined the crystal structure of PGK1 to determine whether the interaction affected the PGK1-related enzyme activities and metabolic events (Fig. 6A). We also designed several mutant forms of PGK1 through site-directed mutagenesis and validated their phenotypes by PGK1 induction (Fig. 6B). Mutant forms with altered phosphoglycerate kinase activities have been reported, and the protein thermal stability and kinetics of these mutants were found to be suppressed^22^. Therefore, we demonstrated phosphoglycerate kinase activity in the PGK1 overexpression model (Fig. 6C). In addition, we detected the intracellular PGK enzyme activity of each mutant form compared to the wild-type. The crystal structure of PGK1 indicated that K191 was the main site for regulating the production of 3-phosphoglycerate (3-PG) by PGK1. The results revealed that the K191del of PGK1 had a lower enzyme activity than the other forms (Fig. 6D). However, we screened the binding affinity of the mutant forms of PGK1 for HTATSF1. Our results revealed that the wild-type, K191del and D285V forms of PGK1 strongly bound to HTATSF1 and that the migration ability was not inhibited by PGK1 induced in H1355 cells. I253T and V266M of PGK1 showed less interaction with HTATSF1, and their migration abilities were inhibited (Fig. 6E, F). We conclude that even though PGK1 promoted lung adenocarcinoma migration, it relied on the interaction of PGK1 protein–protein binding with HTATSF1 rather on metabolic events.

### Correlations between the expression of PGK1/HTATSF1 and clinical outcome

**Fig. 7:**
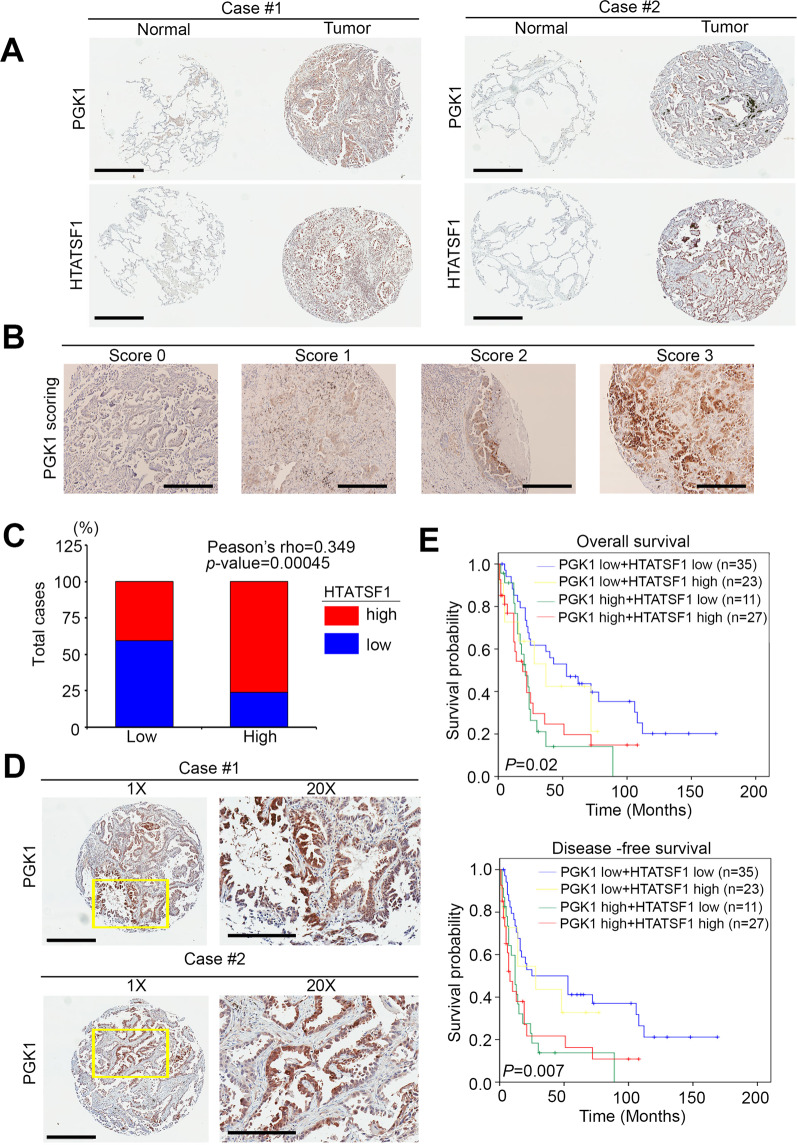
PGK1-HTATSF1 expression as a prognostic factor for clinical lung cancer patients. **A** PGK1 and HTATSF1 protein expression in the paired normal and tumor tissues derived from clinical lung cancer patients. Statistical significance was analyzed by a paired *t*-test. Scale bar: 400 μm. **B** IHC staining for the PGK1 protein. The intensity of IHC staining was scored as a range from 0 to 3. Scale bar: 50 μm. **C** Quantification of HTATSF1 expression by immunohistochemistry analysis of lung cancer specimens by each corresponding clinical parameter. **D** PGK1 protein expression in several tumor tissues derived from clinical lung cancer patients. Scale bar: 400 μm. 20x: 200μm.**E** Kaplan–Meier analysis of the overall survival and disease-free survival probabilities of clinical lung cancer patients according to the intensity (low = 0 and 1, high = 2 and 3) of IHC staining for the PGK1 protein combined with the HTATSF1 protein level.

Next, we performed immunochemical analysis to detect the protein level of PGK1 in the lung cancer tissue array. According to the staining of paired tumor/normal tissues from clinical patients, we observed that a high expression of PGK1 commonly occurred in tumor tissues but was rare in normal adjacent tissues (Fig. 7A). From a series of tissue section slides, our results also revealed that PGK1 expression positively correlated with HTATSF1 in patient specimens (Fig. 7A). Quantitation of IHC staining also showed a higher staining percentage and intensity of PGK1/HTATSF1 in the tumor samples than in the normal group (Fig. 7B). We also validated that more patients had a high level of HTATSF1 expression in the PGK1 overexpression group (Pearson’s *ρ* = 0.349, *P* = 0.00045) (Fig. 7C). In addition, we observed the consistent trends in several references that PGK1 had translocated from the cytoplasm to the nucleus in our tissue array (Fig. 7D)^23,24^. After IHC scoring, the high level of PGK1 combined with the high level of HTATSF1 staining significantly correlated with a poor overall survival and disease-free survival compared with a low level of PGK1 combined with a low level of HTATSF1 in lung cancer (Fig. 7E).

According to the significant correlation between PGK1 and HTATSG1 expression and N stage from our IHC staining results, an estimation of patient survival by the Kaplan–Meier method and log-rank test was also performed. Patients with high PGK1/HTATSF1 expression levels were significantly more likely to have a poorer disease-specific survival (*P* = 0.011) and disease-free survival (*P* = 0.004; Table 1 and Supplementary Table 3). Univariate and multivariate analyses were performed for disease-specific survival and progression-free survival with a Cox proportional hazards regression model (Supplementary Table 2). For disease-specific survival, all parameters, including high PGK1/HTATSF1 expression, higher T stage, higher N stage, and higher M stage were significantly correlated with a decreased disease-specific survival in the univariate analyses. However, only high PGK1/HTATSF1 expression (hazard ratio [HR] = 1.529; 95% confidence interval [CI] = 1.145–2.042; *P* = 0.004) and higher N stage (HR = 2.073; 95% CI = 1.181–3.637; *P* = 0.011) remained independent prognostic factors for disease-specific survival in the multivariate analyses (Supplementary Table 4).

**Table 1:** Clinical relevance of PGK1 expression in lung cancer.

Characteristics	PGK1 expression, *n* (%)			
	*n*	Low (*n* = 59)	High (*n* = 48)	*P* value
Age				
<65 y ≧65 y	56 51	31(55.4) 28(54.9)	25(44.6) 23(45.1)	0.558
Sex				
Male	48	30(62.5)	18(37.5)	0.118
Female	59	29(49.2)	30(50.8)	
Smoking status				
No	40	22(55.0)	18(45.0)	0.570
Yes	67	37(55.2)	30(44.8)	
Histological type				
Adenocarcinoma	65	35(53.8)	30(46.2)	0.669
Squamous carcinoma	35	19(54.3)	16(45.7)	
Large cell carcinoma	7	5(71.4)	2(28.6)	
Stage^a^				
I + II	46	30(65.2)	12(30.0)	0.052
III + IV	61	29(47.5)	26(46.4)	
Tumor status				
T1 + T2	75	41(54.7)	34(45.3)	0.526
T3 + T4	32	18(56.3)	14(43.7)	
Lymph node status				
N0	38	27(71.1)	11(28.9)	0.012^*^
N1-3	69	32(46.4)	37(53.6)	
Distal metastasis status				
M0	79	45(57.0)	34(43.0)	0.338
M1	28	14(50.0)	14(50.0)	

*SD* standard deviation.

^*^p value < 0.05 was considered statistically significant (Student’s *t* test for continuous variables and Pearson’s chi-square test for variables).

^a^The tumor stage, tumor, lymph node, and distal metastasis status were classified according to the international system for staging lung cancer.

## Discussion

Dysregulated metabolism is an emerging hallmark of cancer^25^. However, little is known about the metabolic requirements during cancer progression. Therefore, we utilized in-silico data mining to identify key metabolic genes associated with lung cancer metastasis. Functional overexpression and complementary knockdown were assayed to reveal the identified gene phenotypes in metastasis. Clinical correlations of the identified genes were evaluated to determine their prognostic value in lung cancer patients. Here, we found that phosphoglycerate kinase 1 (PGK1) up-regulation highly correlated with migratory/invasive activity of lung cancer cells and poorer outcomes in clinical lung cancer patients. Unlike previous investigations, we demonstrated that this novel function of PGK1 in cancer was not limited to chemotherapy/radiotherapy^26–29^ or to triggering autophagy^30,31^ in tumorigenesis. Chen et al.^32^ have described that several metabolic-related enzymes, including PGK1, plat roles in lung adenocarcinoma. However, previous results have claimed that there are multiple driver mutation genes and various carcinogens in Western countries (tobacco) and Asia countries (cigarettes). Therefore, we utilized immunohistochemical screening methods for glycolytic enzymes, focusing on patients in Taiwan. We further examined how the expression levels of PGK1 correlated with several genetic alteration events, including EGFR mutants, KRAS mutants, and ALK fusions in our clinical cohort. These findings were validated by IHC staining with a PGK1 antibody in clinical lung cancer tissues. Significantly, the knockdown of PGK1 inhibited, while the forced expression of exogenous PGK1 promoted, the in vitro migratory activity of lung cancer cell lines that had highly or poorly expressed PGK1 protein, respectively. Importantly, PGK1 could serve as an independent prognostic factor and positively correlated with recurrence and poor survival rates in the lung cancer patient cohorts. In recent years, the dysfunction of glycolytic enzymes involved in various cancer phenotypes has been revealed. We investigated the role of PGK1 in lung tumorigenesis, and found that PGK1 was involved in migration and also regulates metastasis. However, the detailed mechanism of this glycolytic enzyme was still unknown except for its enzyme function. Thus, we proposed that PGK1 promoted the cancer phenotype through protein–protein interaction or post-translational modification^33^. Moreover, we identified that PGK1 translocated into difference organelles^23^. Therefore, our results showed that PGK1 can translocate to the nucleus and bind to HTATSF1.

Based on previously investigations, multiple growth factor signaling pathways, including the MEK-ERK pathway^34^, the FOXD3/miR-146 axis^35^, and the PI3K/AKT-mTOR pathway^36,37^, have been shown to cross-talk with alternative glucose metabolic networks. These signals induce a high degree of ATP turnovers in glycolysis and changes in the tumor microenvironment. However, we investigated whether these glycolytic enzymes had nonenzymatic functions in promoting several phenotypes in cancer progression. These undisclosed mechanisms can be found in cancer research through protein-protein interactions or posttranslation modifications of target genes. These studies also suggest that strong side effects or the disruption of metabolic processes in human body should be observed closely during the development of small therapeutic compounds^38^. In recent years, HTATSF1 has been identified as a molecule involved in RNA metabolism that has an increased the expression level during bon metastasis from primary site^22,39^. Besides, we also found that PGK1 and HTATSF1 both upregulated in metastasis animal models^40^. HTATSF1 is also considered to be a cofactor for the stimulation of transcriptional elongation by HIV-1 Tat. Crosstalk and common signatures in HIV and cancer are bound to play an important role. However, little is known in cancer research^41^.

For future research, we will design a specific peptide to block the potential binding sites of the PGK1 and HTATSF1 interaction. From recent studies, glycolysis-related enzymes may act through protein-protein interaction binding or translational modification with other molecules by their enzyme-independent functions^13,42^. In some cases, PKM2 could translocate from the cytoplasm to the nucleus to then regulate the activity of several transcription factors in tumorigenesis^43^. Moreover, aldolase directly binds to γ-actin and controls the cell cytoskeleton and migration regardless of enzyme activity. Immunofluorescence staining detected that HTATSF1 localizes to the nucleoplasm by analysis of The Human Protein Atlas website^44^ (Supplementary Fig. S9). Therefore, we isolated the cytoplasmic and nuclear fractions and demonstrated that PGK1 was translocated from the cytoplasm to the nucleus during overexpression (Fig. 5C). We also proposed that PGK1 may have additional roles in tumorigenesis through interaction with molecules to regulate several phenotypes or the permanent environment for cancer cells, both in metabolic abundancy and deprivation statuses^45,46^. We will postulate and evaluate the potential binding energy and, angles and perform virtual screening. We will show that peptides or candidate compounds inhibit migration ability in vitro and lung nodule formation ability in vivo. Importantly, PGK1 and its interaction partners have served as an independent prognostic factor and positively correlated with patient survival rate and recurrence status in lung cancer patients.

We also comprehensively analyzed the role of PGK1 in several cancer types. In addition to lung cancer or brain tumors from previous studies, we observed that PGK1 was significantly associated with overall and disease-free survival in breast cancer patients. PGK1 has been regarded as one prognostic factor that may correlate with chemoresistance in breast cancer^29^ (Supplementary Fig. S10). Although the binding affinity of PGK1 and HTATSF1 needs to be further assessed in breast cancer. Based on our analysis, PGK1 may be an independent prognostic factor for lung cancer and breast cancer. Furthermore, blocking the interaction between PGK1 and HTATSF1 is a novel option for a clinical therapeutic application.

## Supplementary information

Supplementary Table S1-S4

Supplementary Table S1

Supplementary Table S2

Supplementary figure legends

Supplementary Figure S1

Supplementary Figure S2

Supplementary Figure S3

Supplementary Figure S4

Supplementary Figure S5

Supplementary Figure S6

Supplementary Figure S7

Supplementary Figure S8

Supplementary Figure S9

Supplementary Figure S10
