# Supplementary material for: Metabolic protein phosphoglycerate kinase 1 confers lung cancer migration by directly binding HIV Tat specific factor 1
Source: Cell Death Discov. 2021 Jun 5;7:135. doi: 10.1038/s41420-021-00520-1 (PMC8179927; doi:10.1038/s41420-021-00520-1)
Supplement: Supplementary file 1 — Supplementary Table S1-S4 [file 41420_2021_520_MOESM1_ESM.docx]

**Supplementary information**

**Metabolic Protein Phosphoglycerate Kinase 1 Confers Lung Cancer Migration by Directly Binding HIV Tat Specific Factor 1**

Yu-Chan Chang^1,2^, Ming-Hsien Chan^3^, Chien-Hsiu Li^3^, Chih-Jen Yang^4,5^, Yu-Wen Tseng^3^, Hsing-Fang Tsai^3^ and Michael Hsiao^3,6^

**Supplementary legends**

**Supplementary Table S1.** List of glycolytic related genes and their fold changes in CL1-5 cells compared with CL1-0 cells from GSE42407.

**Supplementary Table S2.** List of interaction partners of PGK1 or PGK2 from BioGrid website.

|  | HTATSF1 expression, n(%) | | | | | |
| --- | --- | --- | --- | --- | --- | --- |
| Characteristics | | n | Low  (n =46) | High  (n =50) | *P* value |  |
| Age | |  |  |  |  |  |
| ＜65y  ≧65y | | 50  46 | 23(52.0)  23(69.6) | 27(48.0)  23(30.4) | 0.695 |  |
| Sex | |  |  |  |  |  |
| Male | | 51 | 27(66.7) | 24(33.3) | 0.343 |  |
| Female | | 45 | 19(53.3) | 26(46.7) |  |  |
| Smoking status | |  |  |  |  |  |
| No | | 44 | 17(54.5) | 27(45.5) | 0.094 |  |
| Yes | | 52 | 29(65.4) | 23(34.6) |  |  |
| Histological type | |  |  |  |  |  |
| Adenocarcinoma | | 57 | 29(50.9) | 28(49.1) | 0.673 |  |
| Squamous carcinoma | | 33 | 15(75.8) | 18(24.2) |  |  |
| Large cell carcinoma | | 6 | 2(66.7) | 4(33.3) |  |  |
| Stage^#^ | |  |  |  |  |  |
| I+ II | | 40 | 24(70.0) | 16(30.0) | 0.045 |  |
| III+ IV | | 56 | 22(53.6) | 34(46.4) |  |  |
| Tumor status | |  |  |  |  |  |
| T1+ T2 | | 69 | 34(65.2) | 35(34.8) | 0.670 |  |
| T3+ T4 | | 27 | 12(48.1) | 15(51.9) |  |  |
| Lymph node status | |  |  |  |  |  |
| N0 | | 36 | 20(66.7) | 16(33.3) | 0.246 |  |
| N1-3 | | 60 | 26(56.7) | 34(43.3) |  |  |
| Distal metastasis status | |  |  |  |  |  |
| M0 | | 69 | 36(63.8) | 33(36.2) | 0.182 |  |
| M1 | | 27 | 10(51.9) | 17(48.1) |  |  |

**Supplementary Table S3.** Clinical relevance of HTATSF1 expression in lung cancer.

**p* value<0.05 was considered statistically significant (Student’s *t*-test for continuous variables and Pearson’s chi-square test for variables). SD represents the standard deviation. ^#^The tumor stage, tumor, lymph node, and distal metastasis status were classified according to the international system for staging lung cancer.

**Supplementary Table S4.** Univariate and multivariate analyses for PGK1/HTATSF1 expression in lung cancer.

| Cox univariate analysis (OS) | | |  |  |  |
| --- | --- | --- | --- | --- | --- |
| Variables | | Comparison | HR (95% CI) | | *P*-value |
| T |  | T3-T4 vs. T1-T2 | 1.836 (1.092-3.087) | | 0.022 |
| N |  | N1-N3 vs. N0 | 2.381 (1.405-4.033) | | 0.001 |
| M |  | M1 vs. M0 | 2.092 (1.248-3.506) | | 0.005 |
| PGK1+HTATSF1 |  | High vs. Low | 1.455 (1.090-1.943) | | 0.011 |
| Cox multivariate analysis (OS) | | |  |  |  |
| Variables | | Comparison | HR (95% CI) | | *P*-value |
| T |  | T3-T4 vs. T1-T2 | 1.159 (0.654-2.055) | | 0.614 |
| N |  | N1-N3 vs. N0 | 1.956 (1.102-3.473) | | 0.022 |
| M |  | M1 vs. M0 | 1.688 (0.978-2.913) | | 0.060 |
| PGK1+HTATSF1 |  | High vs. Low | 1.377 (1.031-1.838) | | 0.030 |
| Cox univariate analysis (DFS) | | |  |  |  |
| Variables | | Comparison | HR (95% CI) | | *P*-value |
| T |  | T3-T4 vs. T1-T2 | 1.973 (1.192-3.266) | | 0.008 |
| N |  | N1-N3 vs. N0 | 2.482 (1.478-4.168) | | 0.001 |
| M |  | M1 vs. M0 | 1.869 (1.131-3.089) | | 0.015 |
| PGK1+HTATSF1 |  | High vs. Low | 1.535 (1.150-2.049) | | 0.004 |
| Cox multivariate analysis (DFS) | | |  |  |  |
| Variables | | Comparison | HR (95% CI) | | *P*-value |
| T |  | T3-T4 vs. T1-T2 | 1.337 (0.750-2.384) | | 0.326 |
| N |  | N1-N3 vs. N0 | 2.073 (1.181-3.637) | | 0.011 |
| M |  | M1 vs. M0 | 1.476 (0.852-2.556) | | 0.165 |
| PGK1+HTATSF1 |  | High vs. Low | 1.529 (1.145-2.042) | | 0.004 |
