# Supplementary material for: Metabolic protein phosphoglycerate kinase 1 confers lung cancer migration by directly binding HIV Tat specific factor 1
Source: Cell Death Discov. 2021 Jun 5;7:135. doi: 10.1038/s41420-021-00520-1 (PMC8179927; doi:10.1038/s41420-021-00520-1)
Supplement: Supplementary file 4 — Supplementary figure legends [file 41420_2021_520_MOESM4_ESM.docx]

**Supplementary information**

**Metabolic Protein Phosphoglycerate Kinase 1 Confers Lung Cancer Migration by Directly Binding HIV Tat Specific Factor 1**

Yu-Chan Chang^1,2^, Ming-Hsien Chan^3^, Chien-Hsiu Li^3^, Chih-Jen Yang^4,5^, Yu-Wen Tseng^3^, Hsing-Fang Tsai^3^ and Michael Hsiao^3,6^

**Supplementary figure legends**

**Supplementary Figure S1.** Quantification of PGK1 gene expression in normal adjacent tissues and paired tumor parts in lung cancer patients from GSE7670 (n=28).

**Supplementary Figure S2.** Kaplan-Meier analysis of PGK2 RNA expression at concurrently low or high levels by *in silico* analysis at the endpoint of overall survival probability in TCGA lung cancer, adenocarcinoma and squamous cell carcinoma subtypes patients (*p*=0.0047, *p*=9.8e-05 and *p*=0.23, respectively).

**Supplementary Figure S3.** Quantification of PGK2 expression by in silico analysis of lung cancer patients by each corresponding clinical events (EGFR mutant and KRAS mutant).

**Supplementary Figure S4.** Heat map of *PGK1/PGK2* expression by *in silico* analysis of lung cancer cell lines from next-generation sequencing database.

**Supplementary Figure S5.** PGK1 overexpressing stable clone of H1355 cells was determined with the migration ability compared to the vector control stable clone (upper). PGK1 knockdown stable clones of H441 cells were determined with the migration ability compared to the shNon-silence control stable clone (lower). Scale bar: 100μm.

**Supplementary Figure S6.** Kaplan-Meier analysis of the PGK1 interaction partners (EIF4G2, PGD, MFAP1, ENO2 and PGKPS) at concurrently low or high levels by *in silico* analysis at the endpoint of overall survival probability or progression-free survival in TCGA lung cancer patients.

**Supplementary Figure S7.** Quantification of the migration ability of several PGK1 interaction molecules in lung adenocarcinoma cells.

**Supplementary Figure S8.** Loading input and IgG control of Pull-down assay

**Supplementary Figure S9.** Quantification of HTATSF1/PGK1 expression by immunohistochemistry analysis of lung cancer specimens by each corresponding clinical parameter.

**Supplementary Figure S10.** Kaplan-Meier analysis of PGK1 RNA expression at concurrently low or high levels by *in silico* analysis at the endpoint of overall survival probability in TCGA breast cancer, including Luminal A/B, HER2-enriched and Triple-negative subtypes patients.
