# Supplementary figures and images for: Metabolic protein phosphoglycerate kinase 1 confers lung cancer migration by directly binding HIV Tat specific factor 1
Source: Cell Death Discov. 2021 Jun 5;7:135. doi: 10.1038/s41420-021-00520-1 (PMC8179927; doi:10.1038/s41420-021-00520-1)

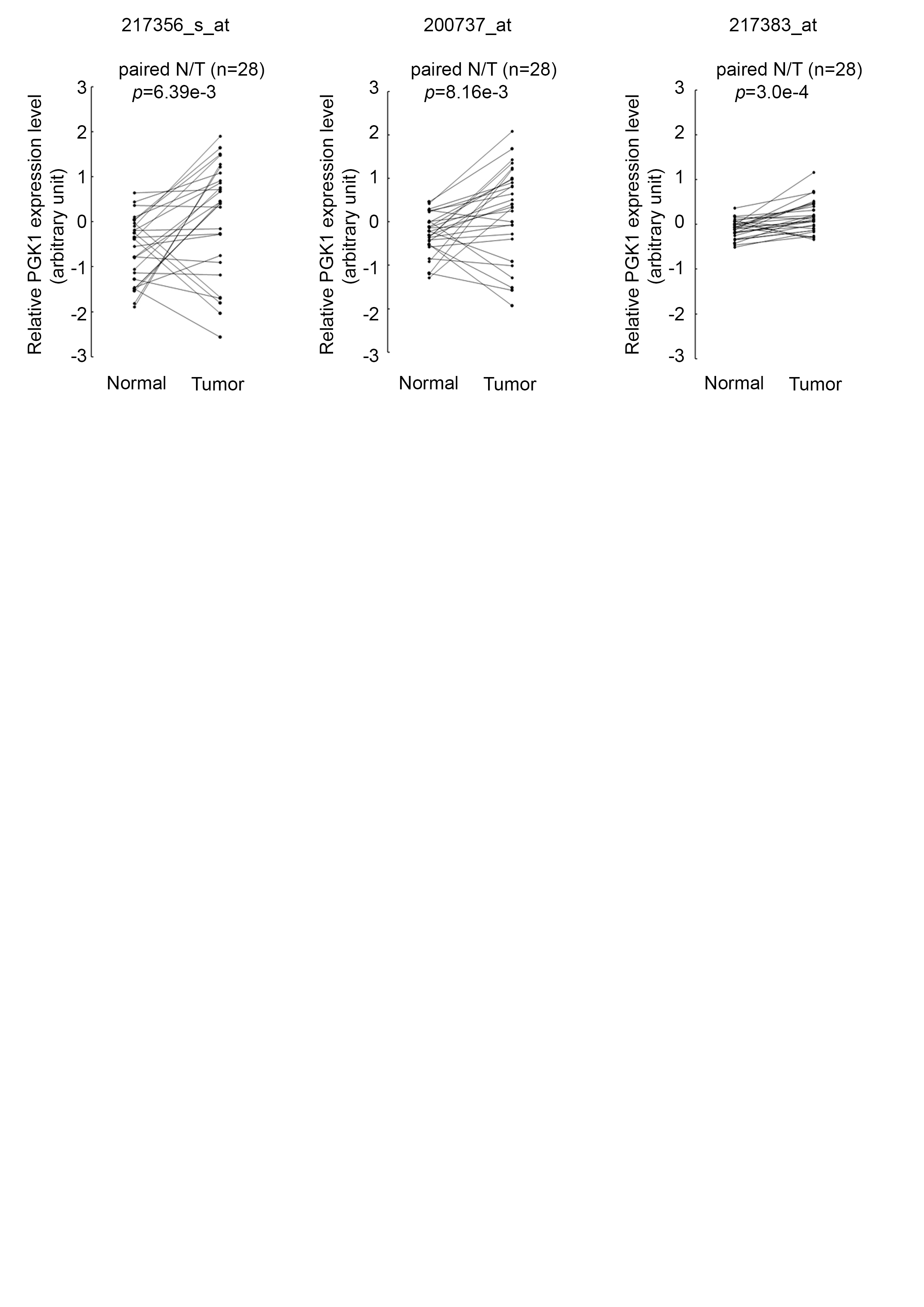

Supplement: Supplementary file 5 — Supplementary Figure S1 [file 41420_2021_520_MOESM5_ESM.tif]

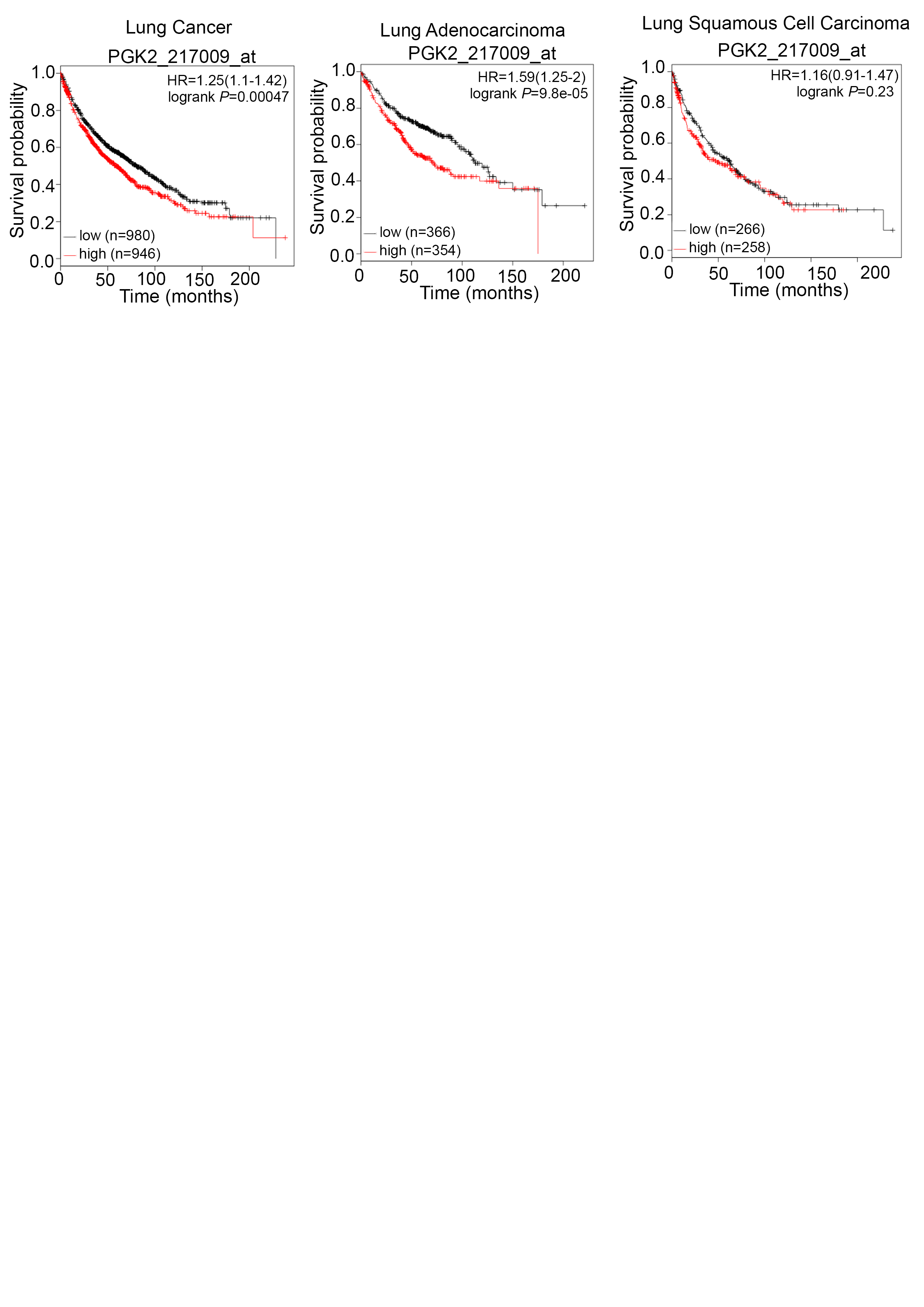

Supplement: Supplementary file 6 — Supplementary Figure S2 [file 41420_2021_520_MOESM6_ESM.tif]

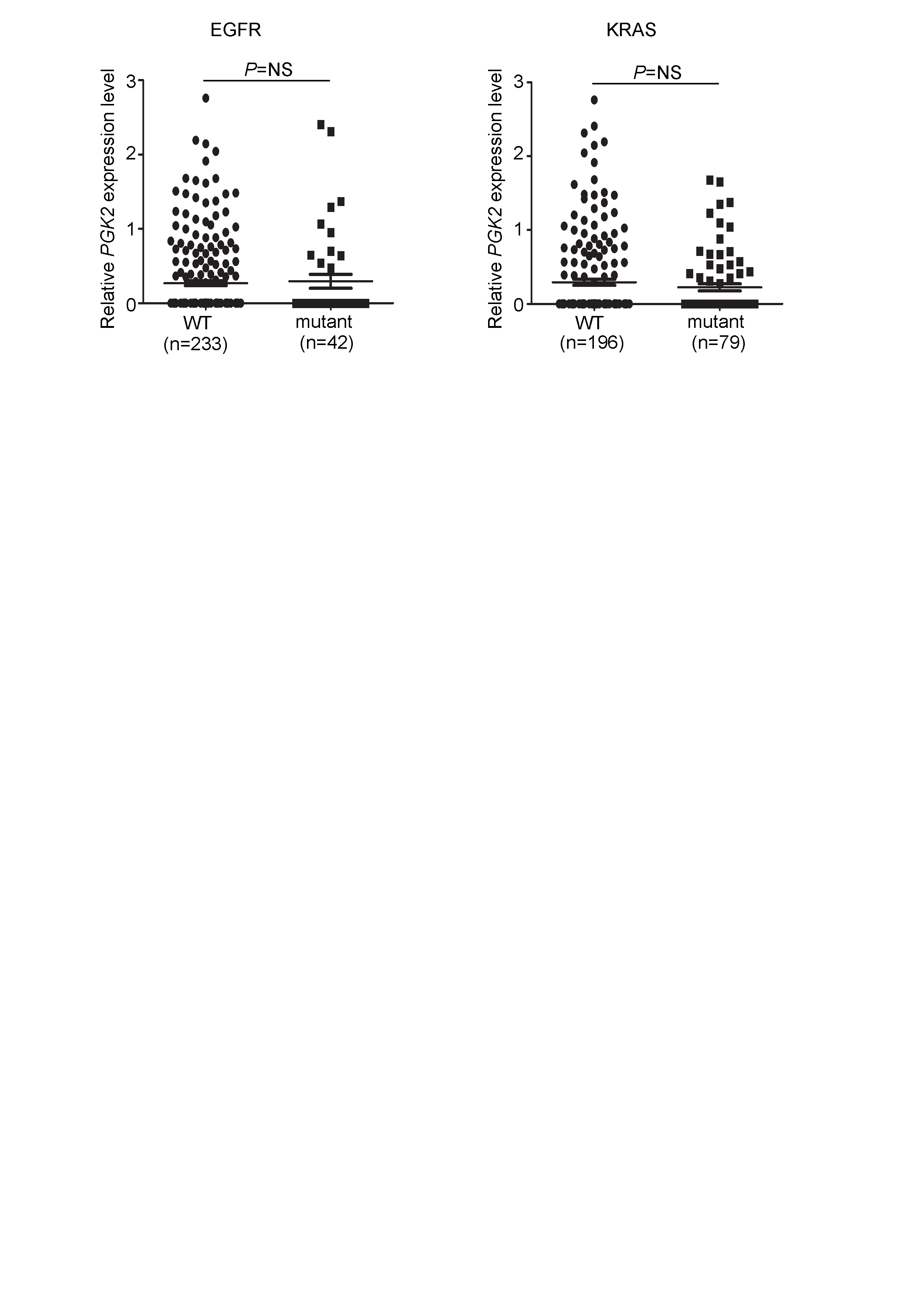

Supplement: Supplementary file 7 — Supplementary Figure S3 [file 41420_2021_520_MOESM7_ESM.tif]

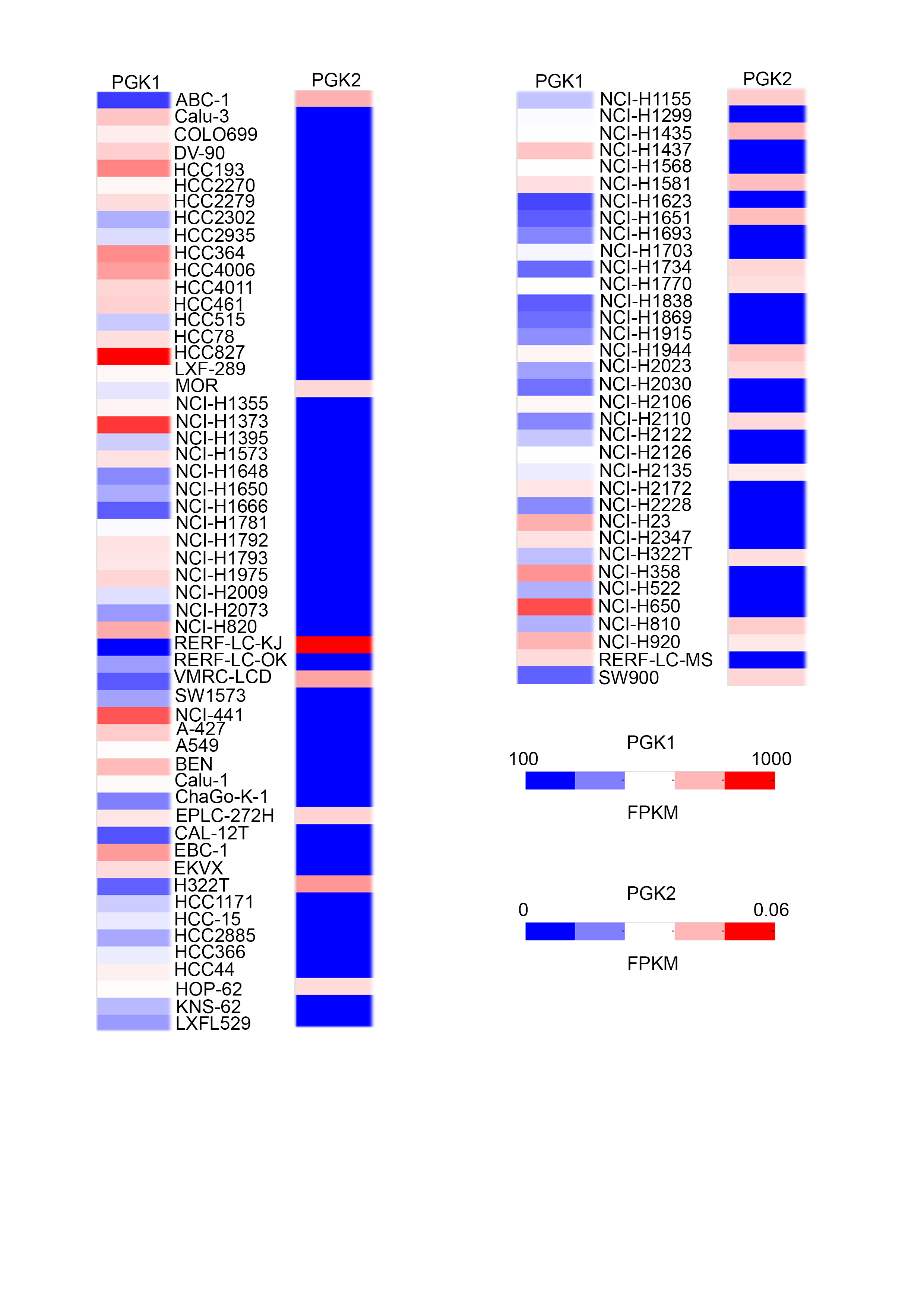

Supplement: Supplementary file 8 — Supplementary Figure S4 [file 41420_2021_520_MOESM8_ESM.tif]

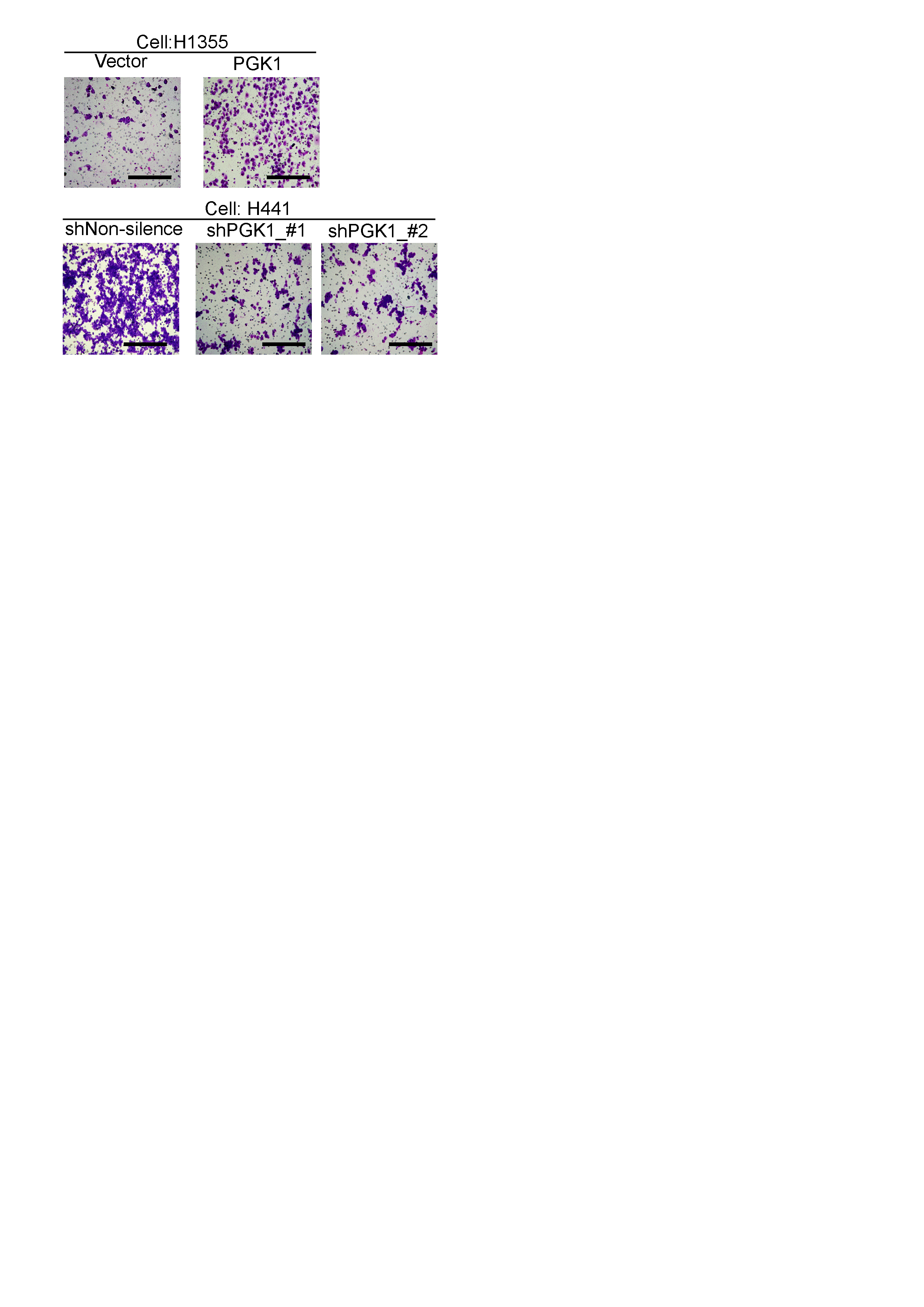

Supplement: Supplementary file 9 — Supplementary Figure S5 [file 41420_2021_520_MOESM9_ESM.tif]

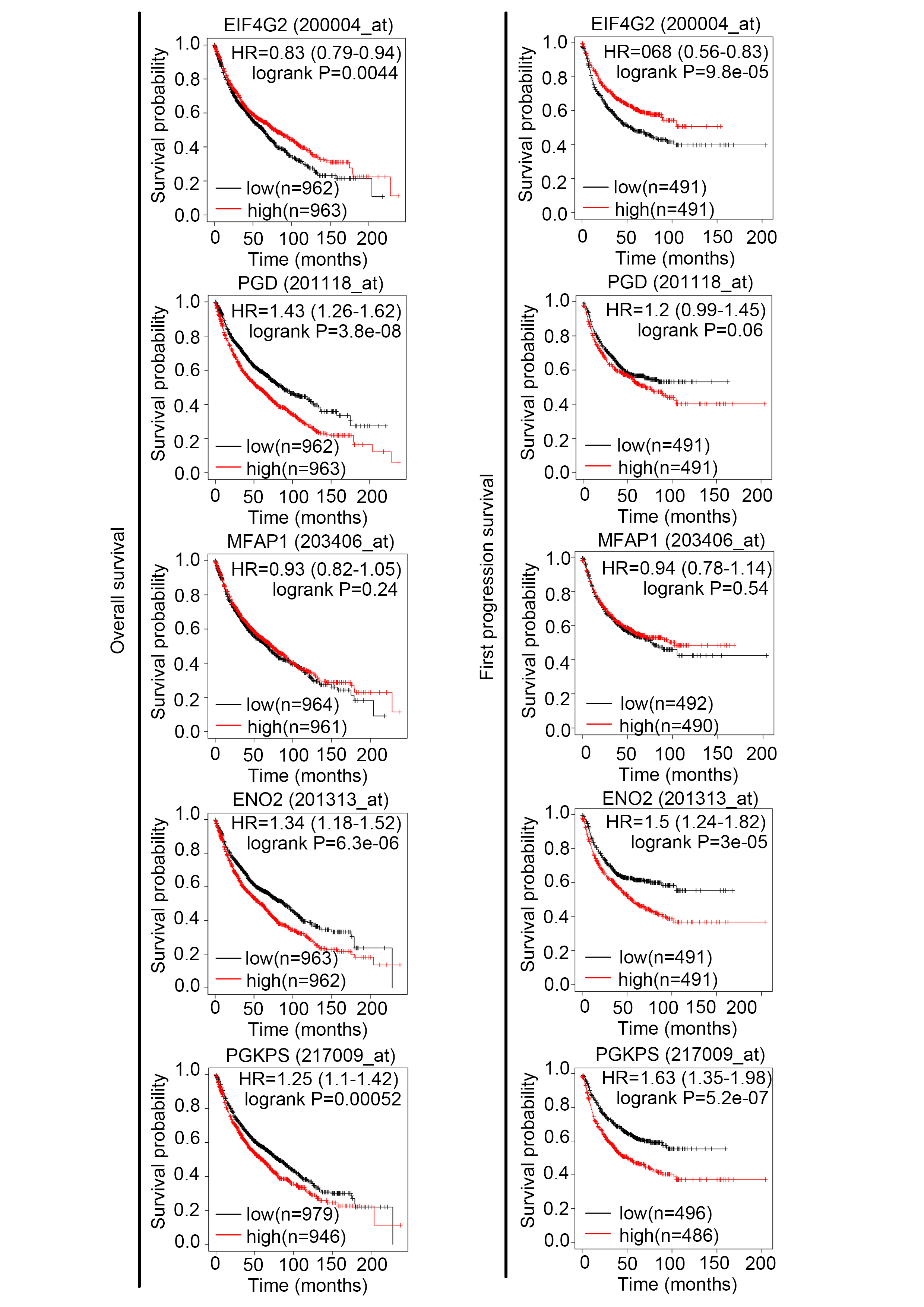

Supplement: Supplementary file 10 — Supplementary Figure S6 [file 41420_2021_520_MOESM10_ESM.tif]

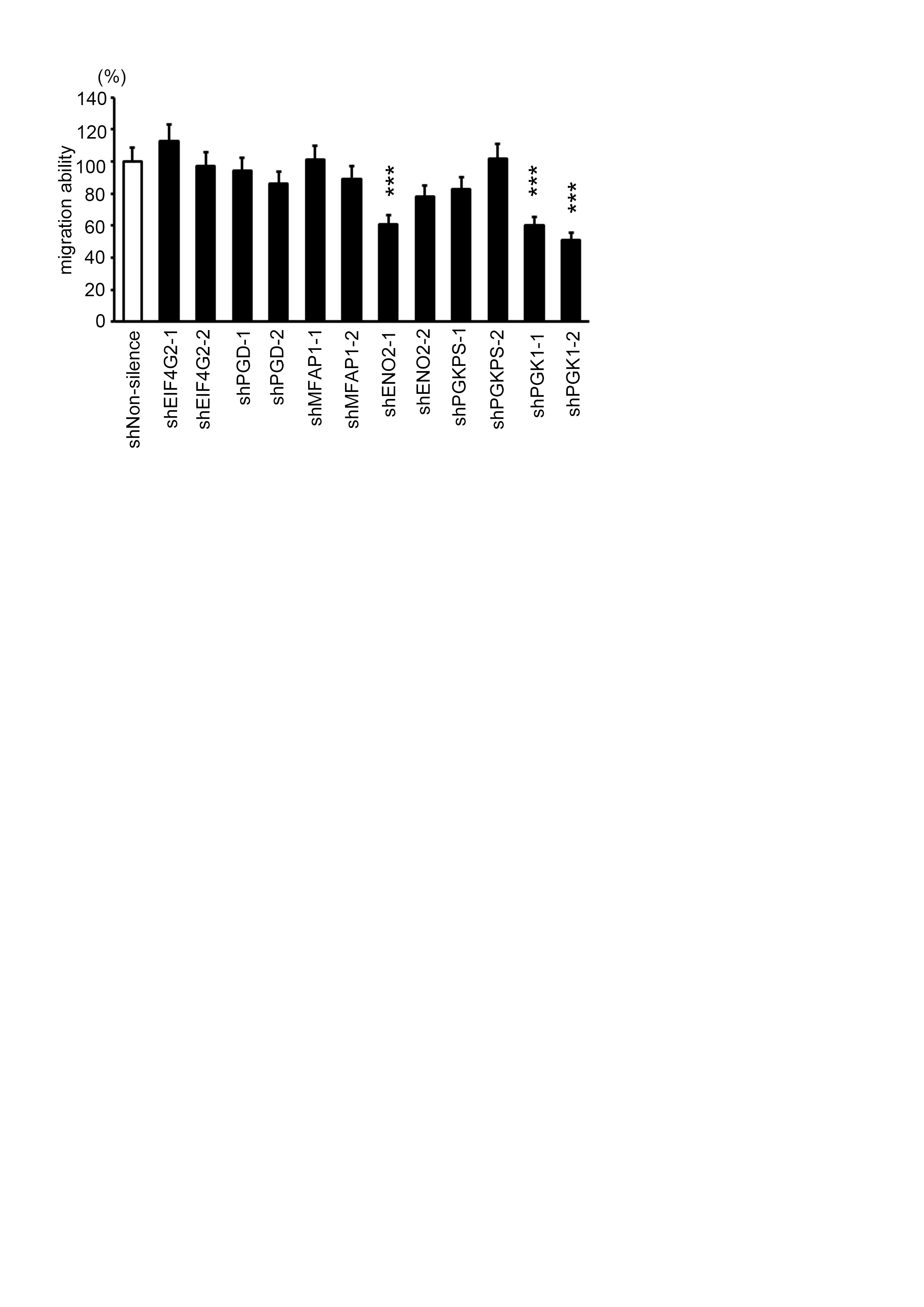

Supplement: Supplementary file 11 — Supplementary Figure S7 [file 41420_2021_520_MOESM11_ESM.tif]

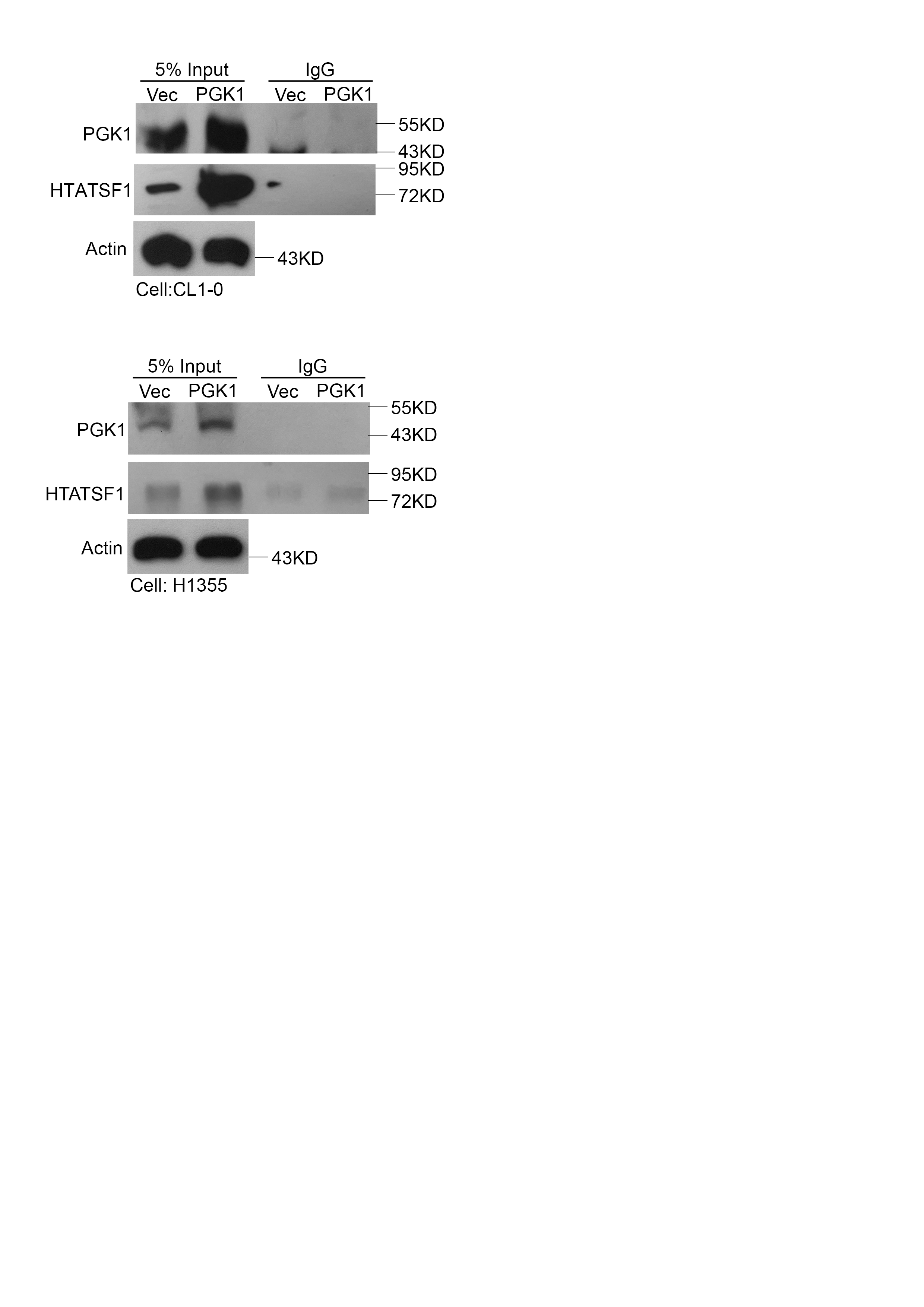

Supplement: Supplementary file 12 — Supplementary Figure S8 [file 41420_2021_520_MOESM12_ESM.tif]

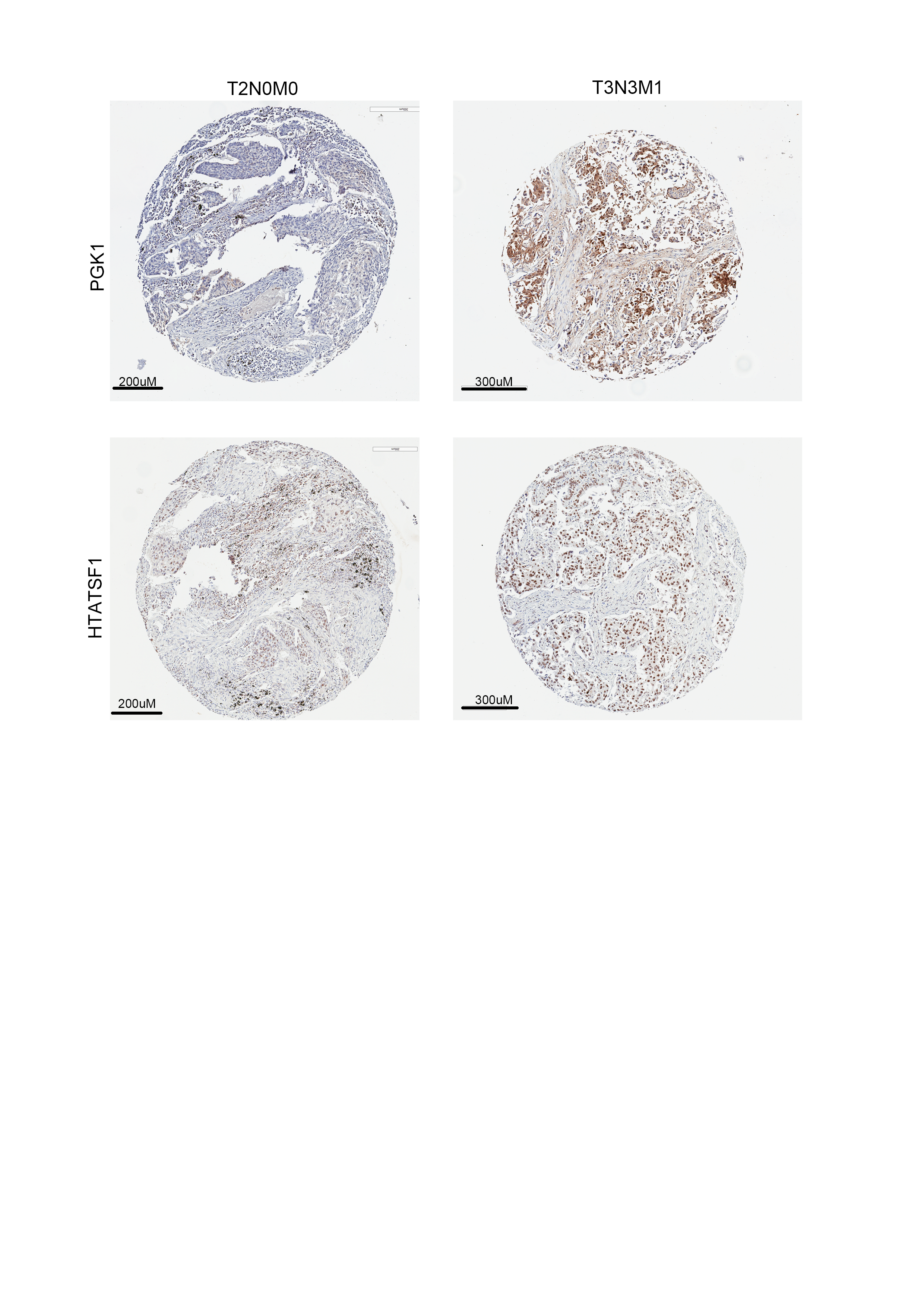

Supplement: Supplementary file 13 — Supplementary Figure S9 [file 41420_2021_520_MOESM13_ESM.tif]

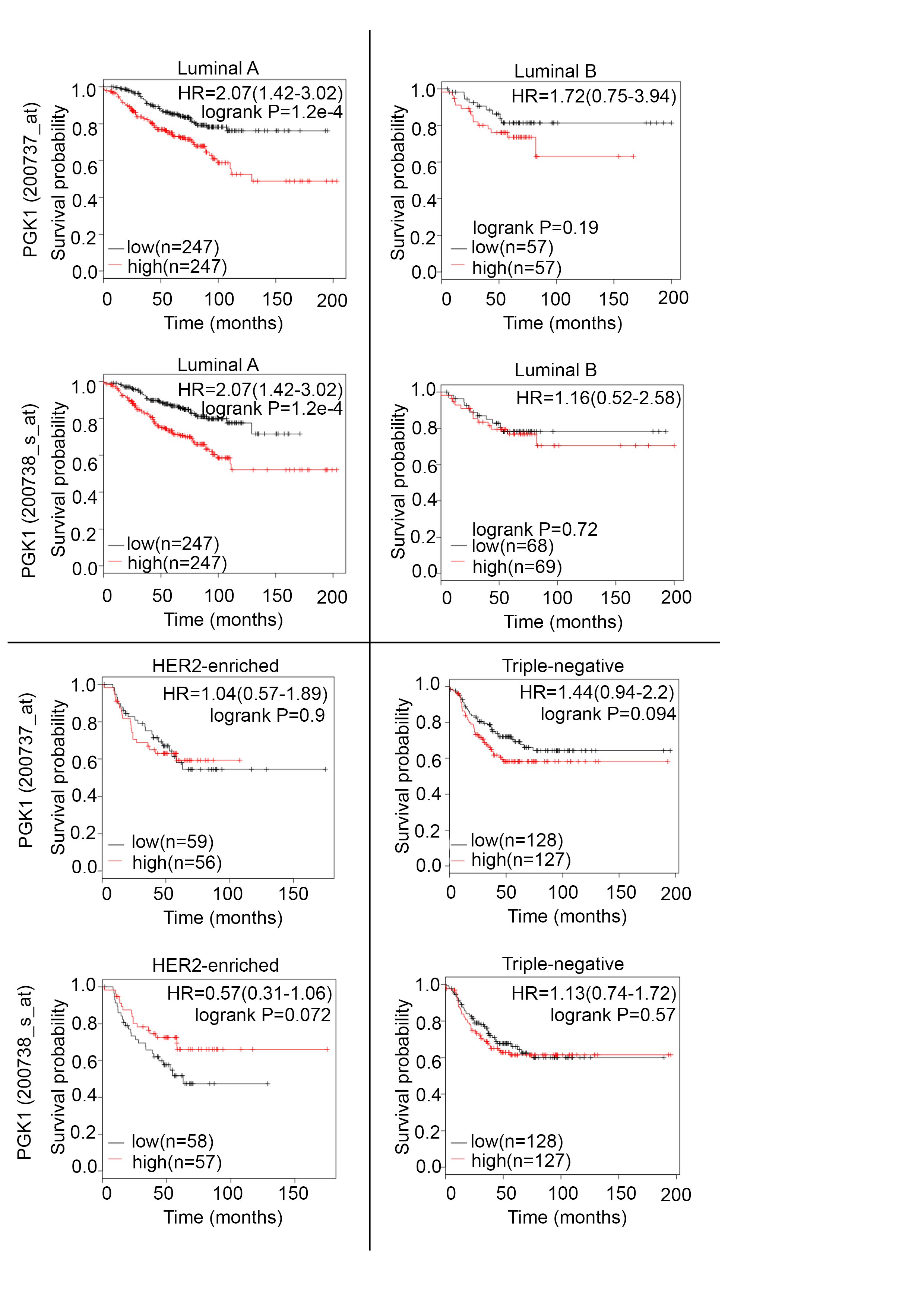

Supplement: Supplementary file 14 — Supplementary Figure S10 [file 41420_2021_520_MOESM14_ESM.tif]
